# Disruption of CSF-1 receptor–mediated metal ion homeostasis in the murine brain promotes neurodegenerative disease

**DOI:** 10.1172/JCI200121

**Published:** 2026-02-26

**Authors:** Violeta Chițu, Julia Alvarenga, Wenna Chen, David Reynolds, Yang Liu, Daqian Sun, Anders Sandell, Virginjia Danylaité-Karrenbauer, Per Uvdal, Iran A.N da Silva, Christophe Sandt, Oxana Klementieva, Ulf Johansson, Kavitha Subramanian Vignesh, Zbigniew K. Wszolek, Dennis W. Dickson, Jennifer T. Aguilian, Simone Sidoli, Deyou Zheng, E. Richard Stanley

**Affiliations:** 1 Department of Developmental and Molecular Biology,; 2 Department of Genetics, and; 3 Department of Cell Biology, Albert Einstein College of Medicine, Bronx, New York, USA.; 4 Division of X-ray Photon Science, Department of Physics and Astronomy, Uppsala University, Uppsala, Sweden.; 5 Department of Clinical Neuroscience, Karolinska Institutet, Stockholm, Sweden.; 6 Department of Neurology, Karolinska University Hospital, Stockholm, Sweden.; 7 Chemical Physics, Department of Chemistry,; 8 NanoLund,; 9 Lund Stem Cell Center, and; 10 Wallenberg Centre for Molecular Medicine, Lund University, Lund, Sweden.; 11 Synchrotron SOLEIL, Saint-Aubin, France.; 12 Department of Experimental Medical Science and; 13 MAX IV Laboratory, Lund University, Lund, Sweden.; 14 Division of Infectious Diseases, College of Medicine, University of Cincinnati, Cincinnati, Ohio, USA.; 15 Department of Neurology and; 16 Department of Neuroscience, Mayo Clinic, Jacksonville, Florida, USA.; 17 Department of Pathology,; 18 Department of Biochemistry, and; 19 The Saul R. Korey Department of Neurology and the Dominick P. Purpura Department of Neuroscience, Albert Einstein College of Medicine, Bronx, New York, USA.

**Keywords:** Cell biology, Neuroscience, Demyelinating disorders

## Abstract

Dominant-inactivating mutations in the colony stimulating factor-1 receptor (*CSF1R*) cause CSF-1R–related leukoencephalopathy (CRL), an adult-onset neurodegenerative disease that is modeled in the *Csf1r^+/–^* mouse. CRL is caused by microglial dysfunction. However, the primary microglial deficit is unknown. To address this question, we employed single-nucleus RNA sequencing of brains from young *Csf1r^+/–^* mice without pathological or behavioral alterations. Reduction of CSF-1R signaling caused metal ion accumulation in brain macrophages, with concomitant activation of cell death and stress response pathways in oligodendrocytes and neuronal subpopulations. Reduction of metallothionein 1 (*Mt1*) and 3 (*Mt3*) gene expression was a common feature in glial and neuronal cells of *Csf1r^+/–^* mice. Overexpression of *Mt1* restored metal ion homeostasis, normalized ROS production in microglia, and prevented the development of behavioral deficits, while *Mt3* deletion had disease-enhancing effects. These findings demonstrate CSF-1R regulation of metal ion homeostasis via metallothioneins in the brain.

## Abstract 2

Reduced levels of a macrophage growth factor receptor disrupts metal ion homeostasis in the brain leading to neurodegeneration

## Graphical abstract

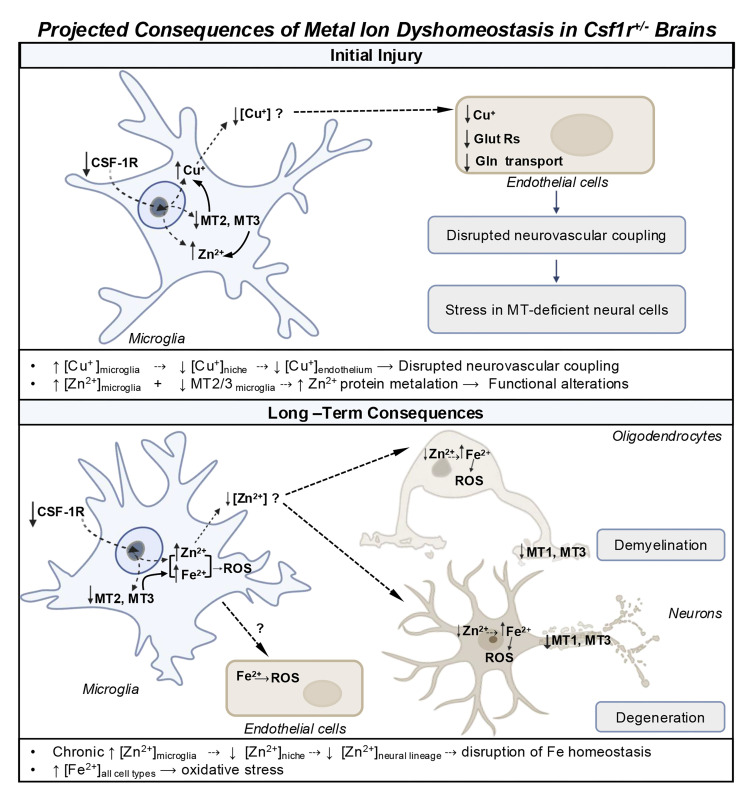

## Introduction

Colony stimulating factor-1 receptor (CSF-1R) is a receptor tyrosine kinase that regulates the maturation of blood monocytes and the differentiation, proliferation, and survival of several populations of tissue macrophages, including microglia (reviewed in ref. 1). In combination with the receptor activator of nuclear factor-κB (aka TNFRSF11A) (2), CSF-1R also regulates osteoclastogenesis and bone resorption (3, 4). Not surprisingly, mutations that inhibit CSF-1R kinase activity affect skeletal development and brain homeostasis to an extent proportional to the severity and allelic expression of the mutation (reviewed in ref. 5).

CSF-1R–related leukoencephalopathy (CRL) is an adult-onset autosomal-dominant disease caused by monoallelic mutations in *CSF1R* that inhibit kinase activity or lead to nonsense-mediated RNA decay (6, 7). CRL is characterized by cognitive impairment, motor dysfunction, psychiatric disorders, and seizures. The disease is incurable and, after diagnosis, progresses continuously with an average time from symptom onset to incapacitation of 4 years and a mean time to death of 7 years (8). The hallmark histopathological features of CRL are bilateral cerebral white matter abnormalities, enlargement of lateral ventricles, and thinning of the corpus callosum. Longitudinal studies show that white matter changes can occur before symptom onset and become more severe as the disease progresses (reviewed in ref. 9).

Histological examination of autopsied brains reveals white matter degeneration and the presence of dilated axons (axonal spheroids) and of lipid- and iron-laden, pigmented macrophages in the affected areas (10). The pigment was identified as ceroid, an end product of severe cellular oxidative damage. The presence of other markers of oxidative damage, such as 4-hydroxynonenal, malondialdehyde, and nitrotyrosine, in the affected tissues was also reported (10). Interestingly, oxidative stress can contribute to axonal spheroid formation through alteration of ion transport (11). Together, these data suggest that oxidative stress, possibly originating in microglia, has an important role in CRL pathology (reviewed in ref. 12).

Most of the *CSF1R* mutations found in CRL impair kinase activity, while others cause nonsense-mediated RNA decay, establishing *CSF1R* haploinsufficiency as a cause of the disease (reviewed in ref. 5). Our laboratory has validated the *Csf1r^+/–^* mouse as a model of CRL that reproduces the hallmark behavioral and histopathological features of early disease, including demyelination, neurodegeneration, and altered microglia (reviewed in ref. 5). Molecular and genetic evidence indicate that CRL is a prototypical primary microglial leukoencephalopathy, as (a) CRL is caused by mutations in the CSF-1R, which in turn is crucial for microglial development and function (13); (b) although CSF-1R is also expressed in neural precursors (14), specific monoallelic targeting in the mononuclear phagocyte lineage is sufficient to produce the behavioral and histopathological changes associated with global heterozygosity, establishing microglia dysfunction as the primary cause of disease (15); (c) evidence of altered microglia function has been provided both in the animal model (16–18) and in patients with CRL (19–21); and (d) inhibition of microglial activity is protective in the mouse model of disease (16, 18, 22, 23). However, because research has largely been restricted to symptomatic cases, the primary microglial deficit leading to disease caused by insufficient CSF-1R signaling is currently unknown. In the present study, we have employed single-nucleus RNA sequencing (snRNA-seq) of brains from *Csf1r^+/–^* mice without pathological or behavioral alterations, in conjunction with genetic, metallomic, behavioral, and cellular studies, to address this question. We demonstrate that insufficient CSF-1R signaling causes dysregulation of metal ion homeostasis, which contributes to the development of CRL.

## Results

### snRNA-seq reveals that Csf1r heterozygosity primarily affects microglia but also neural and endothelial lineage cells in presymptomatic mice.

**Figure 1:**
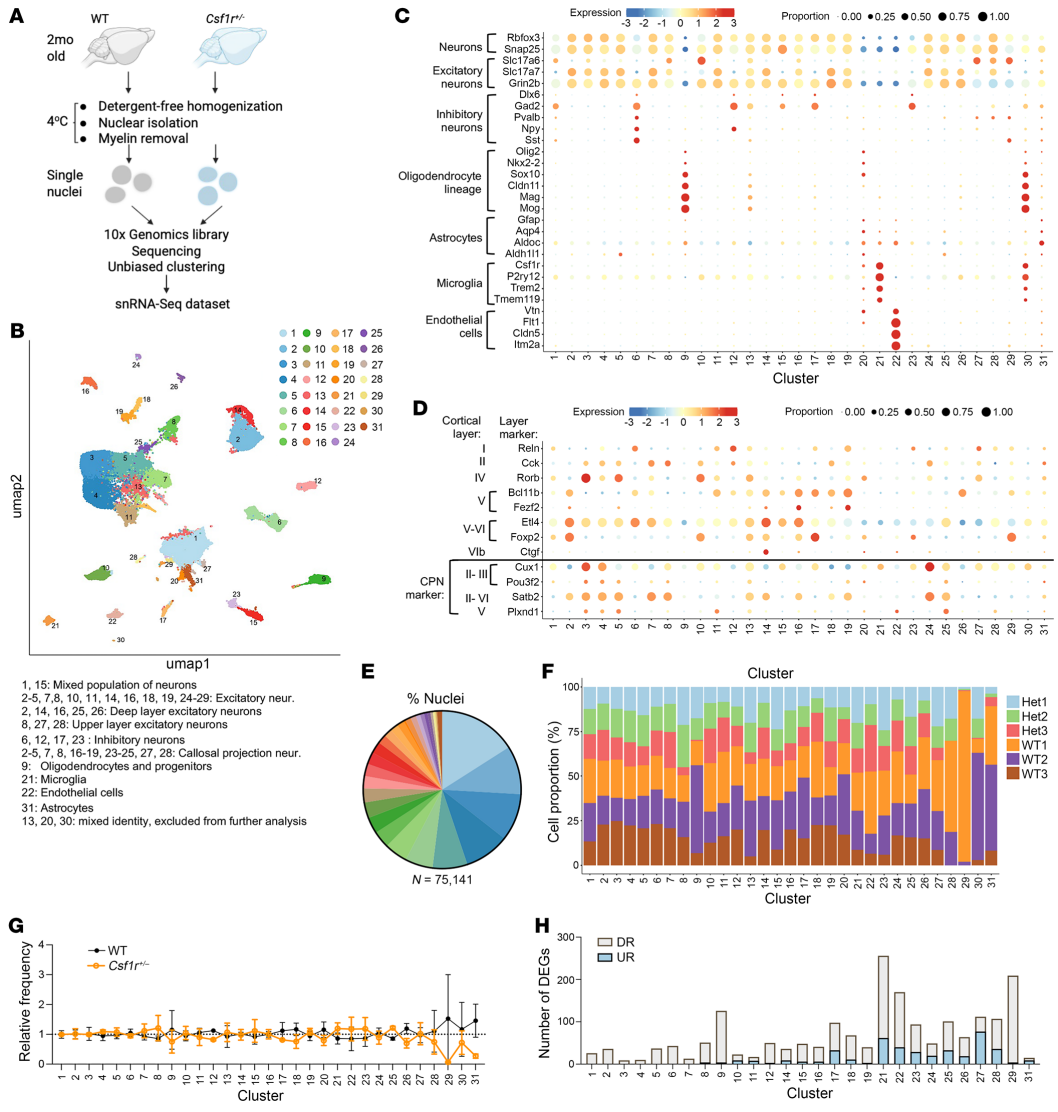
snRNA-seq identifies major populations of neurons and glial cells and shows selective reduction of neuronal subpopulations in *Csf1r^+/–^* mice. (**A**) Diagram of the snRNA-seq workflow. (**B**) Uniform manifold approximation and projection (UMAP) plot showing the clustering of nuclei. *n* = 3 mouse brain samples per genotype; 75,141 total nuclei. (**C**) Dot plot showing the expression of cell type–specific markers. (**D**) Dot plot showing the expression of neuronal markers of cortical layers and callosal projection neurons (CPN). (**E**) Pie chart showing the frequency of each cluster. (**F**) Relative frequencies of WT and *Csf1r^+/–^* nuclei in each cluster. No statistically significant change was detected. Two-way ANOVA, Bonferroni. (**G**) Relative frequency of clusters in WT and *Csf1r^+/–^* brains normalized to the overall frequency in **E**. (**H**) Number of differentially expressed genes (DEGs) in each cluster. The data were obtained from 3 mice/genotype. UR, upregulated; DR downregulated in *Csf1r^+/–^*.

To investigate how reduced CSF-1R signaling affects microglia function and their communication with other brain cells, we performed a snRNA-seq study of brains isolated from 2-month-old asymptomatic *Csf1r^+/–^* mice and wild-type (WT) controls (3 mice per genotype) (Figure 1A). A total of 75,141 individual nuclei were analyzed (44,570 WT and 30,571 *Csf1r^+/–^*) after quality controls. The individual samples were processed and then integrated by the scDAPP pipeline (24), generating 31 clusters (Figure 1B). Cell types in these clusters were identified manually, based on the expression of widely accepted markers (Figure 1, B–E). Most clusters contained transcripts for the pan-neuronal markers *Rbfox3* and *Snap25*. Based on the expression of excitatory or inhibitory neuron markers, these neuronal clusters were classified as excitatory neurons (clusters 2–5, 7, 8, 10, 11, 14, 16, 18, 19, and 24–29), inhibitory neurons (clusters 6, 12, 17, and 23), or mixed population neurons (clusters 1 and 15) (Figure 1, B and C, and Supplemental Table 1; supplemental material available online with this article; https://doi.org/10.1172/JCI200121DS1). Although excitatory neurons were spread among many clusters, we chose not to merge them to allow the identification of clusters enriched in layer V and in callosal projection neurons, which are primarily affected in CRL (15, 25–27). Examination of the expression of cortical layer markers and markers of callosal projection neurons (Figure 1D) revealed that deep layer (V–VI) excitatory neurons were enriched in clusters 2, 14, 16, 25, and 26, with cluster 26 containing mostly Bcl11b^+^ layer V excitatory neurons. Callosal projection excitatory neurons were present in clusters 2–5, 7, 8, 16, 19, 24, and 25. Clusters 9 and 21 were identified as oligodendrocyte lineage and microglia, respectively, while cluster 22 contained endothelial cells. Astrocyte markers were found in clusters 20 and 31 (Figure 1C). However, cluster 20 contained a significant proportion of nuclei expressing oligodendrocyte, microglia, and endothelial cell markers and was omitted from further analysis. Clusters 13 and 30 contained high fractions of nuclei with low detected transcripts and no clear markers and were also excluded from further analysis.

As expected for mice without pathological changes in the brain, most clusters were similarly represented in both genotypes, with some degree of variation among replicates (Figure 1, F and G, Supplemental Table 2). To reduce false positive discoveries, we applied pseudobulking methods (pooling cells of the same mouse) for differential gene expression analysis in each cluster and selected significantly changed genes that were expressed by at least 10% of the cells within the cluster, *P* ≤ 0.01, for pseudobulking differential expression analysis. This revealed that while *Csf1r* deficiency preferentially affected gene expression in microglia (cluster 21), oligodendrocytes (cluster 9) and neural lineage (cluster 29) and endothelial (cluster 22) cells were also significantly affected (Figure 1H and Supplemental Table 3). These results, together with previous evidence that mouse CRL is a primary microgliopathy (15), suggest that even before the onset of overt pathology, insufficient *Csf1r* signaling in microglia changes their function, thereby altering the status of neural and endothelial lineage cells.

### Csf1r^+/–^ microglia exhibit transcriptional alterations consistent with anomalies in transition metal homeostasis.

**Figure 2:**
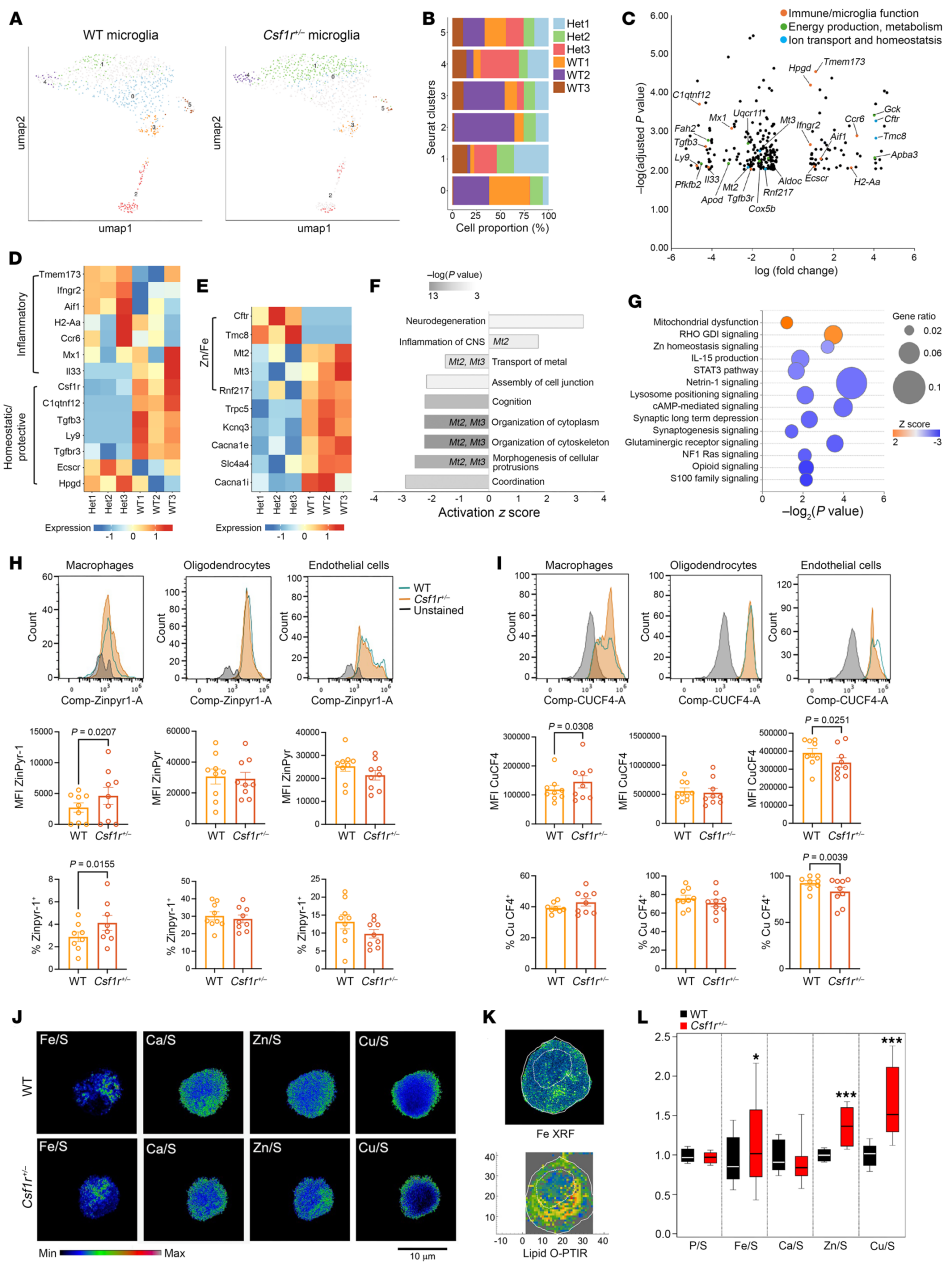
*Csf1r^+/–^* heterozygosity alters mitochondrial function and causes transitional metal ion accumulation in brain macrophages. (**A**) UMAP plot showing the distribution of WT and *Csf1r^+/–^*microglia in individual subclusters. (**B**) Representation of WT and *Csf1r^+/–^* nuclei in each subcluster. (**C**) Volcano plot showing DEGs between WT and *Csf1r^+/–^* microglia, indicating altered expression of transcripts encoding proteins involved in immune function, energy production and metabolism, and ion transport and homeostasis. (**D**) Expression of selected genes that define homeostatic/protective and inflammatory microglial states. (**E**) Examples of dysregulated transcripts related to ion transport, including metallothioneins 2 and 3 (*Mt2* and *Mt3*). (**F**) Ingenuity Pathway Analysis–based (IPA-based) prediction of biological processes affected by *Csf1r* heterozygosity in microglia. The column labels indicate involvement of MTs in the process. (**G**) IPA-based prediction of pathways affected by *Csf1r* heterozygosity in microglia. Orange, activated; blue, inhibited. (**H** and **I**) Distribution of Zn^2+^ (**H**) and Cu^+^ (**I**) in the brains of young (2-month-old) WT and *Csf1r^+/–^* mice. Filled gray curves, unstained control; green lined unfilled curve, WT; filled orange curves, *Csf1r^+/–^*. Each symbol on the chart represents 1 mouse. Means ± SEM; Student’s 1-tailed paired *t* test. Note: Because CD11b is expressed in both microglia and perivascular macrophages, throughout this report we refer to cells isolated by CD11b expression as brain macrophages. However, ~90% of mononuclear phagocytes in the brain are microglia (79). (**J**) Representative XRF images showing the distribution and sulfur content-normalized abundance of Fe, Ca, Zn, and Cu in BMM at steady state in vitro. The color scale indicates the strength of the signal. (**K**) Example of partial colocalization of Fe-enriched (XRF, upper panel) and lipid-rich (OPTIR, lower panel) areas within a cell. The dotted lines delineate the boundaries of the cell and nucleus. (**L**) Quantification of the normalized abundance of Fe, Ca, Zn, and Cu in BMM (WT, black; *Csf1r^+/–^*, red). Each *Csf1r^+/–^* value is normalized to the corresponding WT value (set to 1). Box height: 75% to 25%, whiskers: 1 SD. *P < 0.05, ***P < 0.0005 (Student’s *t* test). Macrophages were obtained from 5 mice/genotype.

As the *Csf1r* is predominantly expressed in microglia and CRL is a primary microglial leukoencephalopathy, we initially studied the impact of *Csf1r* heterozygosity on microglia. At 2 months of age, *Csf1r^+/–^* mice do not exhibit behavioral deficits, and there is no evidence of demyelination or neurodegeneration in the brain (27). Therefore, alterations in the transcriptomic profiles of microglia should reflect deficits caused by *Csf1r* deficiency rather than adaptive responses to an ongoing pathology. Indeed, at this stage, we did not detect the emergence of new populations of microglia (Figure 2, A and B). Furthermore, there was no evidence for upregulation of signature genes for pathogenic microglia, or the downregulation of homeostatic markers (Supplemental Figure 1), both of which have been previously reported to occur in microglia isolated from affected mice (16) and CRL patient postmortem brains (21).

Analysis of the 62 genes upregulated and 194 genes downregulated in *Csf1r^+/–^* microglia revealed enrichment in transcripts related to immunity and inflammation, energy production and lipid metabolism, as well as ion transport (Figure 2, C–E, and zal Supplemental Table 3). Consistent with this, neurodegeneration, inflammation of the CNS, and transport of metal were among the top biological processes predicted to be affected in microglia by *Csf1r* heterozygosity (Figure 2F). Notably, we observed the downregulation of *Mt2* and *Mt3*, which are Zn^2+^-, Cu^+^-, and Fe^2+^-binding proteins (28) and the upregulation of *Cftr*, which mediates the accumulation of zinc in activated macrophages (29) (Figure 2E). Furthermore, IPA predicted an impairment in Zn homeostasis signaling (Figure 2G). Since MTs buffer heavy metals, these data were suggestive of disruption of metal ion homeostasis with accumulation of labile Zn^2+^, Cu^+^, and possibly Fe^2+^ in microglia. To determine the significance of these findings for brain metal ion homeostasis, we investigated how *Csf1r* deficiency affects the distribution of Zn^2+^, Cu^+^, and Fe^2+^ among brain macrophages, oligodendrocytes, endothelial cells, and neurons in vivo, in young mice. As shown in Figure 2, H and I, labile Zn^2+^ and Cu^+^ ion loads were increased in brain macrophages of *Csf1r^+/–^* mice. Furthermore, there was a slight increase in the percentage of macrophages containing labile Fe^2+^ (Supplemental Figure 2). The distribution of Zn^2+^ and Fe^2+^ in oligodendrocytes and endothelial cells was not significantly affected by *Csf1r* insufficiency in young animals (Figure 2H and Supplemental Figure 2). Furthermore, the levels of Fe^2+^, Zn^2+^, and Cu^+^ were unchanged in neurons (data not shown). However, the increased retention of Cu^+^ in macrophages was associated with a decrease in Cu^+^ signals in endothelial cells and a lower percentage of Cu^+^-containing endothelial cells (Figure 2I).

To investigate whether the CSF-1R controls metal ion homeostasis in macrophages in the absence of other cell types, we prepared bone marrow–derived macrophages (BMM) from both WT and *Csf1r^+/–^* mice, differentiated and propagated in vitro, in the absence of stimuli other than CSF-1. We measured the levels of Fe, Ca, Zn, and Cu, normalized to S content, using x-ray fluorescence (XRF) microscopy. Figure 2J shows representative single-cell XRF images that illustrate the abundance and distribution of Fe, Ca, Zn, and Cu. Interestingly, while Ca and Zn were mostly cytosolic, Cu concentrated in the proximity of the plasma membrane (Figure 2J) while Fe exhibited an asymmetric enrichment. Optical photothermal IR microscopy (OPTIR) is a spatial-omics technique based on infrared absorption, which enables label-free molecular profiling of cells and tissues at submicron resolution (30). Direct correlation with lipid infrared (IR) signals obtained with OPTIR prior to XRF suggests a tendency for Fe to overlap with lipid-rich organelles, possibly lysosomes (Figure 2, J and K). Consistent with the ex vivo data, quantification of XRF signals revealed a significant enrichment in Zn, Cu, and Fe content in *Csf1r^+/–^* macrophages differentiated in vitro (Figure 2L).

Together, the data suggest that *Csf1r* deficiency directly causes the accumulation of transition metal ions in brain macrophages. Over time, this may reduce their bioavailability to other cells of the brain.

### Csf1r heterozygosity causes transcriptomic changes consistent with endothelial cell dysfunction and with the activation of stress and cell death pathways in oligodendrocytes and subsets of neurons.

**Figure 3:**
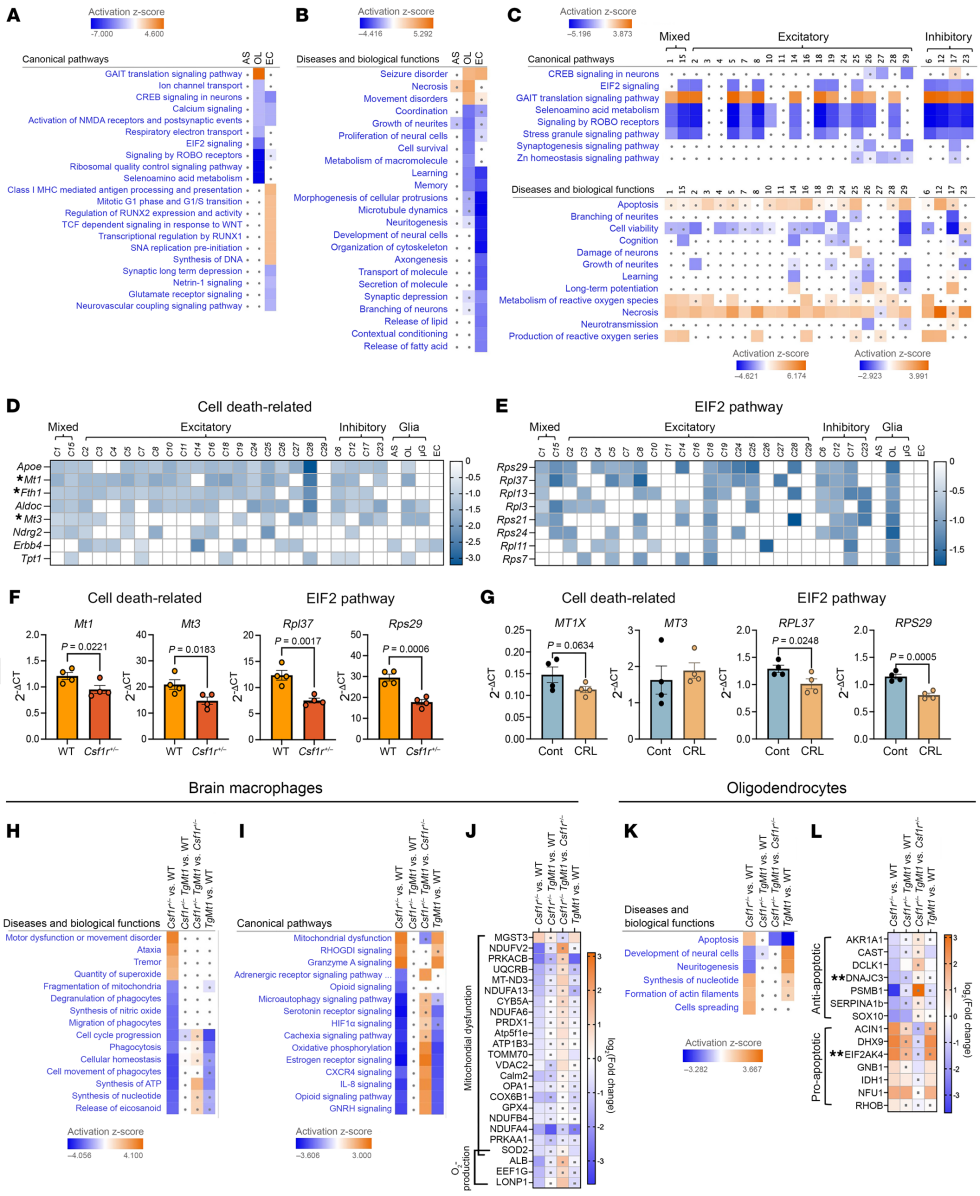
Early activation of stress and cell death pathways in *Csf1r^+/–^* mice and effects of transgenic overexpression of MT1. (**A** and **B**) IPA-based prediction of pathways (**A**) and biological processes (**B**) affected in glial and endothelial lineage cells of young *Csf1r^+/–^* mice. (**C**) IPA-based prediction of pathways (upper panel) and biological processes (lower panel) affected in neurons of young *Csf1r^+/–^* mice. The small gray spots indicate lack of significance (*Z* score < 2 and/or *P* > 0.05). (**D**) Heatmap showing the expression of cell death–related transcripts. Asterisks mark the position of *Mt1*, *Fth1*, and *Mt3* gene transcripts encoding proteins involved in metal ion homeostasis. (**E**) Heatmap showing the expression of EIF2 pathway-related transcripts. AS, astrocytes; OL, oligodendrocytes; μG, microglia, EC, endothelial cells. (**F**) qRT-PCR validation of selected changes in gene expression in mouse brains. Means ± SEM; 1-tailed Student’s *t* tests. Each symbol on the chart represents 1 mouse. (**G**) Expression of cell death and EIF2 pathway genes in patients with CRL. Each circle on the charts represents 1 patient. One-tailed Student’s *t* tests. (**H**–**L**) Proteomic analysis of the effects of *Csf1r* heterozygosity and *Mt1* overexpression in brain macrophages and oligodendrocytes of 3-month-old mice. Data from 5 mice/condition. (**H**–**J**) Macrophages. IPA-generated predictions of biological processes (**H**), pathways (**I**), and proteins involved in regulation of mitochondrial function and superoxide production (**J**). (**K** and **L**) Oligodendrocytes. (**K**) IPA-generated predictions of biological processes affected. (**L**) Changes in the expression of anti- and pro-apoptotic proteins. **Proteins involved in the inhibition (DNAJC3) and activation (EIF2AK4) of the integrated stress response.

Next, we examined how disruption of microglial homeostasis by *Csf1r* insufficiency affects neural and endothelial lineage cells. Ranking clusters by the number of differentially expressed genes placed cluster 29, containing excitatory neurons; cluster 22, containing endothelial cells; and cluster 9, containing oligodendrocytes, at the top of the list. In contrast, cluster 31, containing astrocytes, showed a limited number of differentially expressed genes (9 upregulated, 6 downregulated), and IPA failed to identify biological processes or pathways significantly affected by these gene expression changes (Figure 3, A and B).

Prompted by the perivascular pattern of myelin loss (10) and angiopathy (31, 32) observed in autopsied brain tissue from patients with CRL, previous studies suggested that disruption of endothelial/microglial crosstalk and vascular dysfunction might contribute to CRL pathology (reviewed in ref. 5). Consistent with this, we observed that in *Csf1r^+/–^* mice free of myelin and neuronal pathology, there are significant gene expression changes in endothelial cells (Figure 1H). Analysis of the gene expression changes revealed downregulation of transcripts encoding glutamate receptors (*Gria1*, *Gria4*, *Grid2*, *Grik1*, *Grik2*, and *Grm1*), muscarinic acetylcholine receptors (*Chrm2*, *Chrm3*), *Gabrg3* encoding the GABA-A receptor subunit γ3, and *Ryr3* encoding the ryanodine receptor 3 intracellular calcium channel (Supplemental Table 3 and Supplemental Figure 3). Indeed, IPA confidently predicted the inhibition of both glutamate receptor signaling and neurovascular coupling in endothelial cells of *Csf1r^+/–^* mice (Figure 3A). These changes are suggestive of impaired neurovascular coupling that could restrict blood flow. In addition, alteration in the transendothelial transport of several molecules important for central nervous system function, including upregulation of folate transporter *Slc19a1* and downregulation of glutamine transporter *Slc38a1*, likely impairing glutamine supply, was observed. (Supplemental Figure 3 and Figure 3B). Together, these data suggest that *Csf1r* heterozygosity is associated with perturbations in brain endothelial cell function that may affect both cerebral blood flow and neurotransmitter production and that precede the onset of overt pathology.

Among neural lineage cells, multiple neuronal clusters and the oligodendrocytes exhibited transcriptional alterations consistent with altered translation through activation of the GAIT (interferon-γ–activated inhibitor of translation) pathway and inhibition of the eukaryotic initiation factor 2 (eIF2) pathway may reflect a stress response (Figure 3, A, C, and E). While there is currently no evidence that the GAIT translational control system functions in neural lineage cells, inhibition of EIF2 pathway may reflect a stress response.

In silico analysis also predicted the activation of cell death pathways in multiple clusters of excitatory and inhibitory neurons as well as in oligodendrocytes (Figure 3, B and C). However, since there is no histological evidence of cellular death in the brains of 2-month-old *Csf1r^+/–^* mice (15, 27), these transcriptional alterations may reflect the initiation of pathways leading to cell death, rather than its execution. A comparison of cell death–related transcripts commonly downregulated in neurons included *Apoe* and *Aldoc*, which may also indicate disruption of lipid homeostasis and energy production (Figure 3D). Notably, as in microglia, the transcripts encoding Mts were commonly affected in neurons and oligodendrocytes (Figure 3D). In fact, *Mt1* was downregulated in 21 of the 28 clusters examined, while *Mt3* was downregulated in 15 clusters.

To validate these findings, we utilized qRT-PCR to measure the expression of Mts and ribosomal transcripts regulated by the eukaryotic initiation factor 2 (eIF2) pathway in brain tissue. As shown in Figure 3F, the expression of *Mt1*, *Mt3*, *Rpl37*, and *Rps29* was reduced in the white matter of young *Csf1r^+/–^* mice. To determine whether our findings are relevant to human disease, we investigated the expression of the same set of genes in autopsied brain tissue from patients with CRL (Supplemental Table 7). Out of the 8 isoforms of human MT1, *MT1X* tended to be downregulated (*P* = 0.06) in the white matter of CRL, while *MT3* was expressed at normal levels (Figure 3G). The expression of *RPL37* and *RPS29* was significantly decreased, consistent with activation of a ribosomal stress response (33). Together, these data establish that decreased expression of MTs and activation of a stress response are early features of CRL in the mouse that may be also relevant to human disease.

### Pharmacological activation of eIF2B is insufficient to suppress cognitive impairment in Csf1r^+/–^ mice.

Various stressors induce the phosphorylation of the eIF2α subunit, leading to inhibition of eIF2B and a global reduction in both protein synthesis (34) and rRNA transcription (35). To probe the significance of inhibition of the eIF2 pathway, we investigated whether treatment with an allosteric inhibitor of phosphorylated eIF2α, 2BAct (36), would prevent development of disease. WT and *Csf1r^+/–^* mice were fed chow containing 2BAct, or control chow, from 2 months of age. Commencing at 16 months of age, their behavior was evaluated in tests of spatial memory and motor coordination. As shown in Supplemental Figure 4, A and B, the rescue of eIF2B activity was insufficient to suppress cognitive impairment in *Csf1r^+/–^* mice, though it tended to alleviate motor dysfunction (Supplemental Figure 3C). These data suggest that restoration of eIF2 signaling is insufficient to prevent the deterioration of neurological function associated with *Csf1r* heterozygosity.

### Overexpression of Mt1 improves homeostasis in brain macrophages and in oligodendrocytes of 3-month-old Csf1r^+/–^ mice.

Through control of Zn^2+^ and Cu^+^ bioavailability, MTs regulate a series of biological processes, including gene expression, mitochondrial function, and metabolism. They also play a role in oxidative defense, by scavenging ROS and excess labile Cu^+^ and Fe^2+^, which, if left unchecked, can further the production of ROS via the Fenton reaction (37–39). Thus, we hypothesized that if dysregulation of brain metal ion homeostasis contributes to CRL, increased MT expression would be beneficial, whereas decreased expression would exacerbate disease development. In a preliminary experiment, we crossed *Csf1r^+/–^* and *Csf1r^+/+^* mice with *Mt1*-overexpressing (*TgMt1*) mice (40) and examined the effect of increased *Mt1* on microglial and oligodendrocyte protein expression by proteomic analysis, at 3 months of age. Consistent with the transcriptomic data (Figure 2G), the proteomic profiling of *Csf1r^+/–^* brain macrophages (Supplemental Table 4) indicates mitochondrial dysfunction and disruption of oxidative phosphorylation (Figure 3I), both of which could contribute to the predicted decrease in synthesis of ATP and increase in superoxide (Figure 3H). Furthermore, oligodendrocyte proteomic profiles (Fig. 3, K and L; Supplemental Table 5) also corroborate the transcriptomic data (Fig. 3B), both indicating the activation of cell death pathways in oligodendrocytes. Transgenic overexpression of *Mt1* prevents mitochondrial dysfunction, restores oxidative phosphorylation and energy production, and suppresses superoxide generation in *Csf1r^+/–^* macrophages while concomitantly suppressing oligodendrocyte apoptosis (Figure 3, H–L). These results indicated a significant beneficial effect of increased *Mt1* expression on macrophage and oligodendrocyte health in young mice and prompted investigation of the effects of increased *Mt1* expression on disease development.

### Overexpression of Mt1 attenuates and genetic inactivation of Mt3 accelerates the development of behavioral deficits in Csf1r^+/–^ mice.

**Figure 4:**
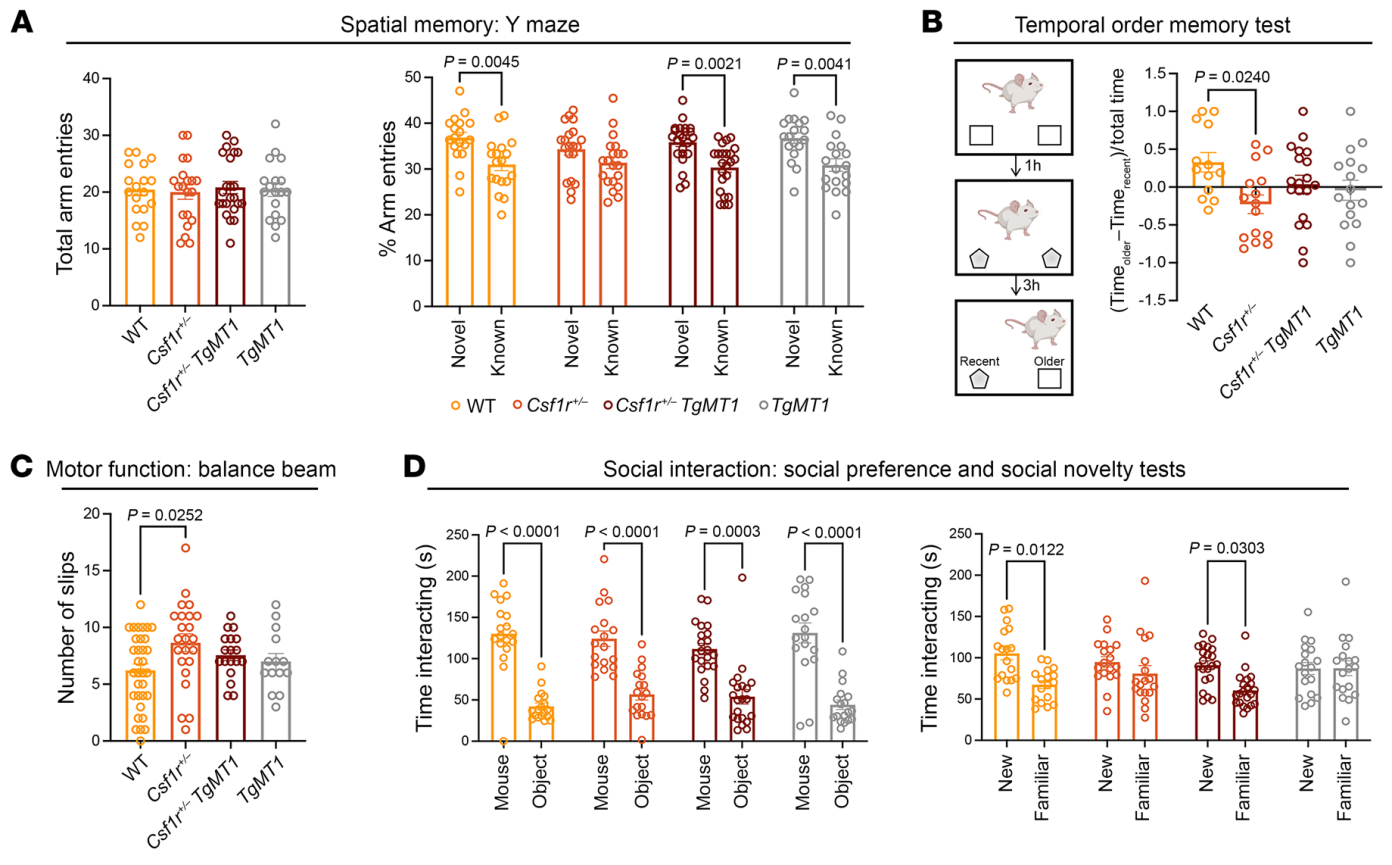
Transgenic overexpression of *Mt1* suppresses symptom development in *Csf1r^+/–^* mice. (**A**) Left panel: No change in total exploratory activity among groups (1-way ANOVA *P* = 0.96). Right panel: Overexpression of *Mt1* prevents the development of spatial memory deficits in *Csf1r^+/–^* mice (2-way ANOVA, Bonferroni). (**B**) Left panel: Schematic of the temporal order test used to assess brain connectivity. Right panel: The temporal order test shows that the overexpression of *Mt1* prevents the impairment of brain connectivity in *Csf1r^+/–^* mice (1-way ANOVA, Tukey’s). (**C**) Attenuation of motor dysfunction in *Csf1r^+/–^* mice by *Mt1* overexpression (1-way ANOVA, Tukey’s). (**D**) Evaluation of social interaction. Left panel: Preferential exploration of mouse compared with object was not affected by *Csf1r* heterozygosity (2-way ANOVA, Bonferroni). Right panel: Loss of preference toward the novel mouse compared with the familiar mouse, observed in *Csf1r^+/–^* mice, was suppressed by *Mt1* overexpression. Right panel: (2-way ANOVA, Bonferroni). Tests were performed starting at 16 months of age. Each circle represents 1 mouse. Means ± SEM. The *P* values are shown only for the statistically significant differences.

To investigate the functional consequences of *Mt1* overexpression, we generated a large cohort of mice comprising *WT*, *Csf1r^+/–^*, *Csf1r^+/–^ TgMt1^/+^*, and *WT TgMt1^/+^* mice and characterized them starting from 16 months of age, when *Csf1r^+/–^* mice are symptomatic. As shown previously (16, 18), aged *Csf1r^+/–^* mice exhibited deficits in hippocampus-dependent spatial memory evidenced by their failure to explore the novel arm in the Y maze task. *Csf1r^+/–^* mice were completely rescued in this task by overexpressing *Mt1* (Figure 4A). To address brain connectivity, we employed the temporal order memory test. Performance in this test depends on a functional interaction between the hippocampus and the perirhinal or medial prefrontal cortices (41). This communication was disrupted in *Csf1r^+/–^* mice, which failed to show an exploratory preference toward the least recently seen object (Figure 4B). This phenotype was alleviated by *Mt1* overexpression. Similarly, the defects in motor coordination were alleviated by overexpressing *Mt1* (Figure 4C). We also explored cerebellar function, using a test for social interaction. As shown previously (18), *Csf1r^+/–^* mice exhibited a lack of preference toward the novel mouse in the social novelty test, which was also fully rescued by *Mt1* overexpression (Figure 4D). These results indicate that *Mt1* overexpression rescues behavioral deficits of *Csf1r^+/–^* previously shown to be critical measures of disease development.

**Figure 5:**
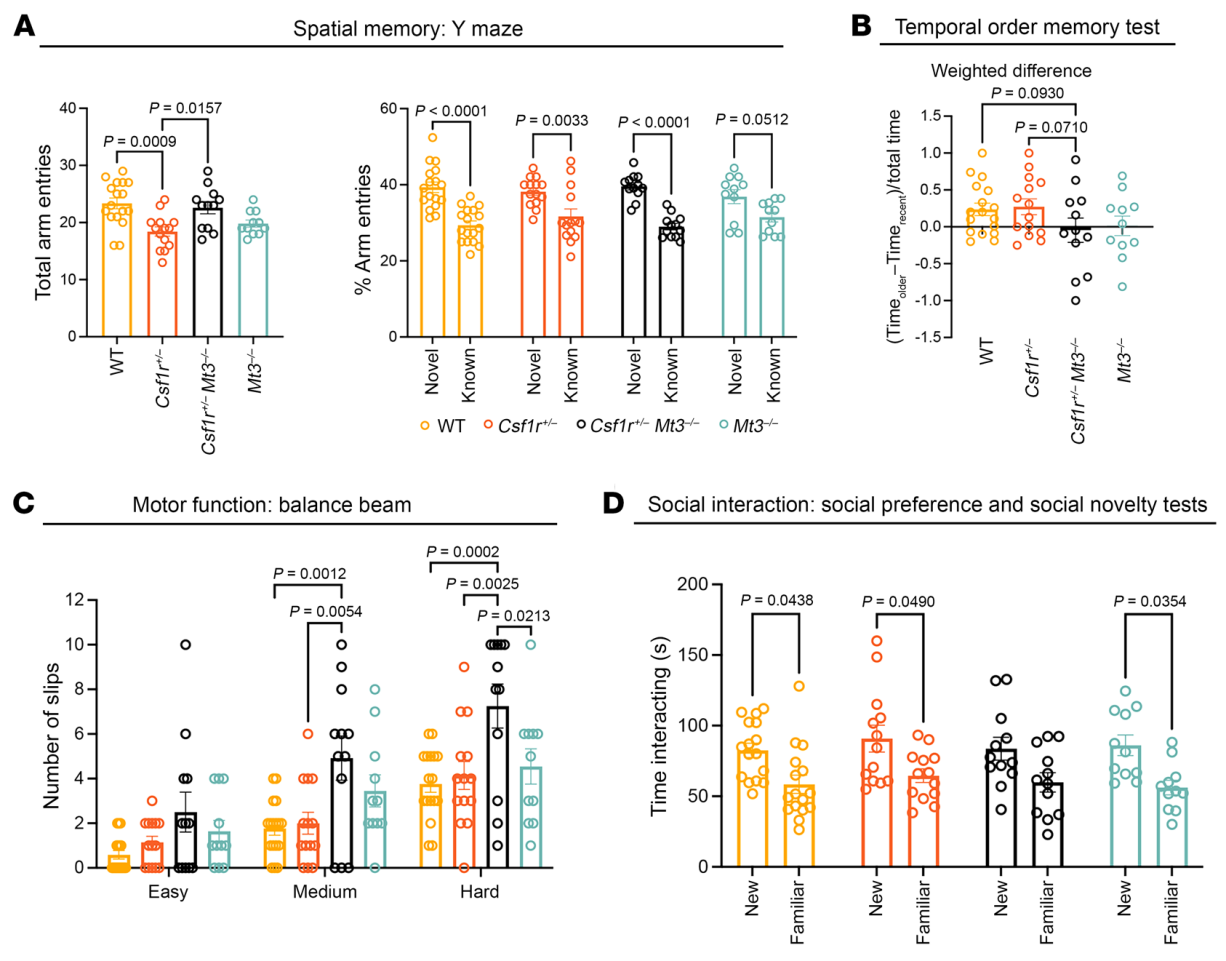
Genetic ablation of *Mt3* accelerates symptom development in *Csf1r^+/–^* mice. Behavioral characterization was initiated at 7 months of age when *Csf1r^+/–^* mice are asymptomatic (Supplemental Figure 5). (**A**) Y maze test of spatial memory. *Csf1r^+/–^* mice exhibited decreased exploratory activity (left panel). However, this finding was inconsistent among cohorts (Supplemental Figure 5). One-way ANOVA, Bonferroni. Right panel, spatial memory is not impaired at this age, regardless of genotype. Two-way ANOVA, Bonferroni. (**B**) Temporal order memory test. Reduced propensity of *Csf1r^+/–^ Mt3^–/–^* mice to explore the least recently seen object suggests a tendency to develop brain connectivity deficits. One-way ANOVA; Fisher least significant differences. (**C**) Balance beam test. *Csf1r^+/–^ TgMt1* mice develop motor deficits earlier than their *Csf1r^+/–^* counterparts. The labels on the abscissa indicate the difficulty of the test, which was inversely proportional to the beam diameter (easy, 2.25 cm; medium, 1.9 cm; hard, 1.6 cm). Two-way ANOVA, Bonferroni. (**D**) Social novelty test. *Csf1r^+/–^ TgMt1* mice develop social interaction deficits earlier than *Csf1r^+/–^* mice. Two-way ANOVA, Bonferroni. Means ± SEM. Each symbol on the chart represents 1 mouse.

We next sought to determine the functional consequences of reduction of MT activity. Despite the already low level of expression of *Mt3* in *Csf1r^+/–^* mice (Figure 3), we tested whether complete elimination of *Mt3* expression would accelerate *Csf1r^+/–^* disease development. We took advantage of *Mt3^–/–^* mice (42) to generate a cohort comprising *WT*, *Csf1r^+/–^*, *Csf1r^+/–^ Mt3^–/–^*, and *Mt3^–/–^* mice, which were functionally evaluated through behavioral testing, starting at 7 months of age, when *Csf1r^+/–^* mice are free of symptoms (Supplemental Figure 5). In contrast with the protective effects of increased MT activity, targeting *Mt3* accelerated the development of brain connectivity, motor coordination, and social interaction deficits in *Csf1r^+/–^* mice (Figure 5).

Overall, these data on MT overexpression and depletion suggest that alteration of metal ion homeostasis plays an important role in the progression of asymptomatic *Csf1r^+/–^* mice to the CRL phenotype.

### Overexpression of Mt1 restores metal ion homeostasis in the brains of aged Csf1^+/–^ mice while Mt3 deficiency exacerbates the accumulation of Fe^2+^.

**Figure 6:**
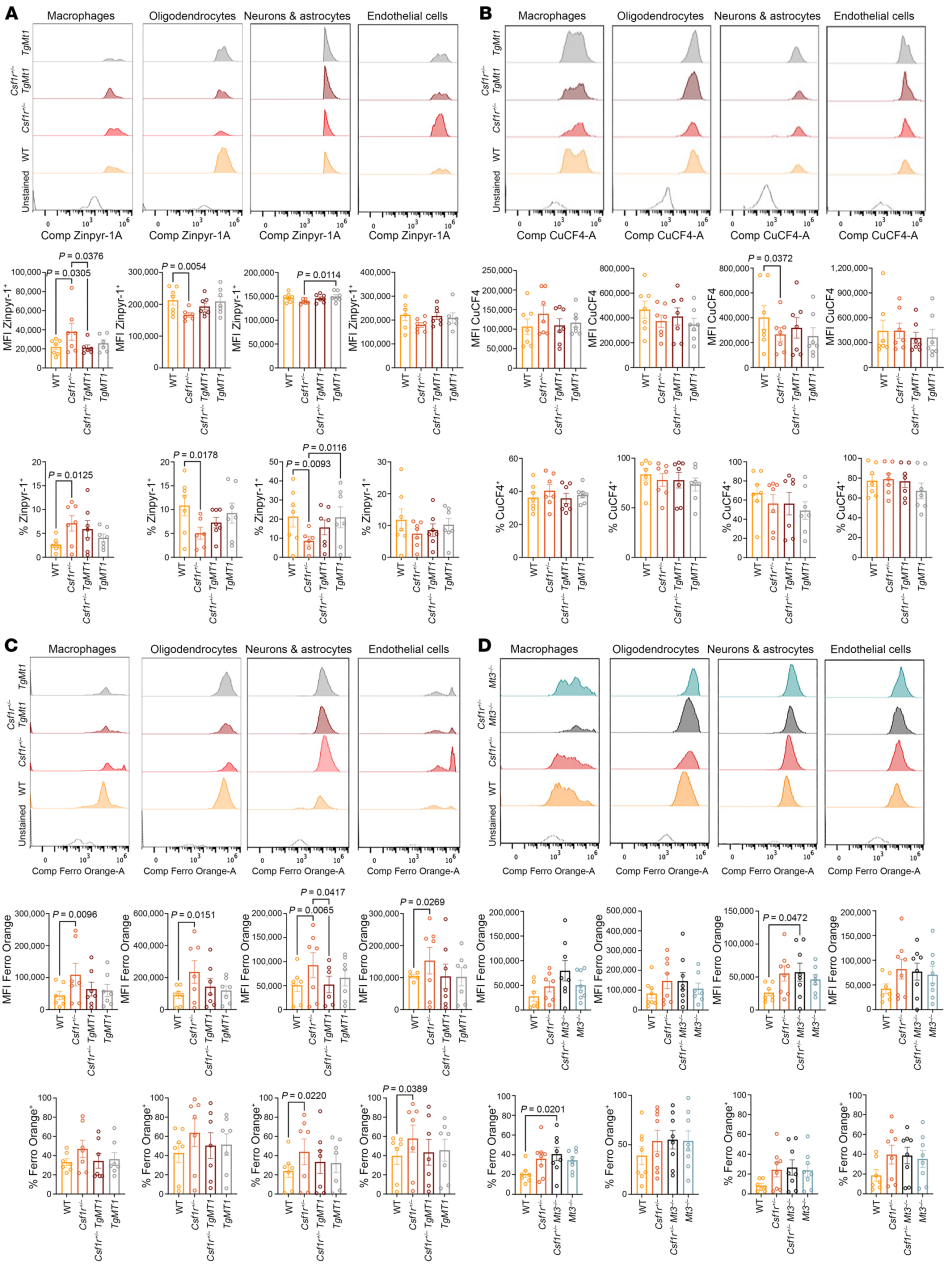
Overexpression of *Mt1* prevents the increase in labile Zn^2+^, Cu^+^, and Fe^2+^ in the brains of *Csf1r^+/–^* mice. (**A**) Measurements of labile Zn^2+^. The accumulation of labile Zn^2+^ in brain macrophages and its decrease in neural lineage cells of aged (>17-month-old) *Csf1r^+/–^* mice are both suppressed by the overexpression of *Mt1*. (**B**) Measurements of labile Cu^+^. Overexpression of *Mt1* attenuates Cu^+^ deficiency in neurons and astrocytes. (**C** and **D**) Measurements of labile Fe^2+^. (**C**) Aged *Csf1r^+/–^* mice exhibit significant accumulation of redox-active Fe^2+^ macrophages, neural lineage cells, and endothelial cells, which is suppressed by the overexpression of *Mt1*. (**D**) *Mt3* deletion exacerbates Fe^2+^ accumulation in brain macrophages and neural lineage cells of presymptomatic (8-month-old) mice. Upper panels, representative histograms; middle panels, MFI; lower panels, percentage positive cells. Each circle represents 1 mouse. Means ± SEM. One-way ANOVA, Dunnett’s post hoc test. The *P* values are shown only for the statistically significant differences.

MTs are present in the cytosol, nucleus, mitochondria, lysosomes, and endosomes, as well as in the extracellular space. They bind Zn^2+^ and deliver it to intracellular compartments, thus controlling its storage and distribution (43). In addition to Zn^2+^, MTs bind redox-active Cu^+^ and Fe^2+^, diminishing their participation in the generation of ROS via the Fenton reaction (28, 38). They can also be secreted from and taken up by other cells, thereby controlling the distribution of their metal ligands in the tissue (44). These properties prompted us to investigate whether overexpression of *Mt1* could restore metal ion homeostasis in the brains of aged mice and thus contribute to the amelioration of pathology. Flow cytometric analysis shows that in aged, symptomatic *Csf1r^+/–^* mice, overexpression of *Mt1* reduces the accumulation of labile Zn^2+^ in macrophages and prevents deficiency in neural lineage cells (Figure 6A). Aged *Csf1r^+/–^* mice also tended to accumulate labile Cu^+^ in macrophages and exhibit reduced levels of labile Cu^+^ in neurons and astrocytes (Figure 6B). However, the most striking phenotype was the significant accumulation of labile Fe^2+^ in all cell types of aged *Csf1r^+/–^* mice, which was suppressed by the overexpression of *Mt1* (Figure 6C).

Next, we addressed the effect of decreasing MT activity on transitional metal homeostasis following genetic inactivation of *Mt3*. Apart from causing a reduction in the levels of labile Zn^2+^ in oligodendrocytes, the absence of MT3 had no significant effect on Zn^2+^ or Cu^+^ homeostasis in the brains of mice at 8–9 months old (Supplemental Figure 6). However, it exacerbated the accumulation of Fe^2+^ in the macrophages and neurons/astrocytes of *Csf1r^+/–^* mice (Figure 6D). Since labile Fe^2+^ has a high propensity to participate in redox reactions that produce detrimental ROS, our data suggest that the earlier disease onset observed in *Csf1r^+/–^ Mt3^–/–^* mice might be related to the early accumulation of Fe^2+^ and oxidative stress–mediated tissue damage.

### Overexpression of Mt1 and deletion of Mt3 have opposite effects on the levels of ROS in Csf1r^+/–^ macrophages.

**Figure 7:**
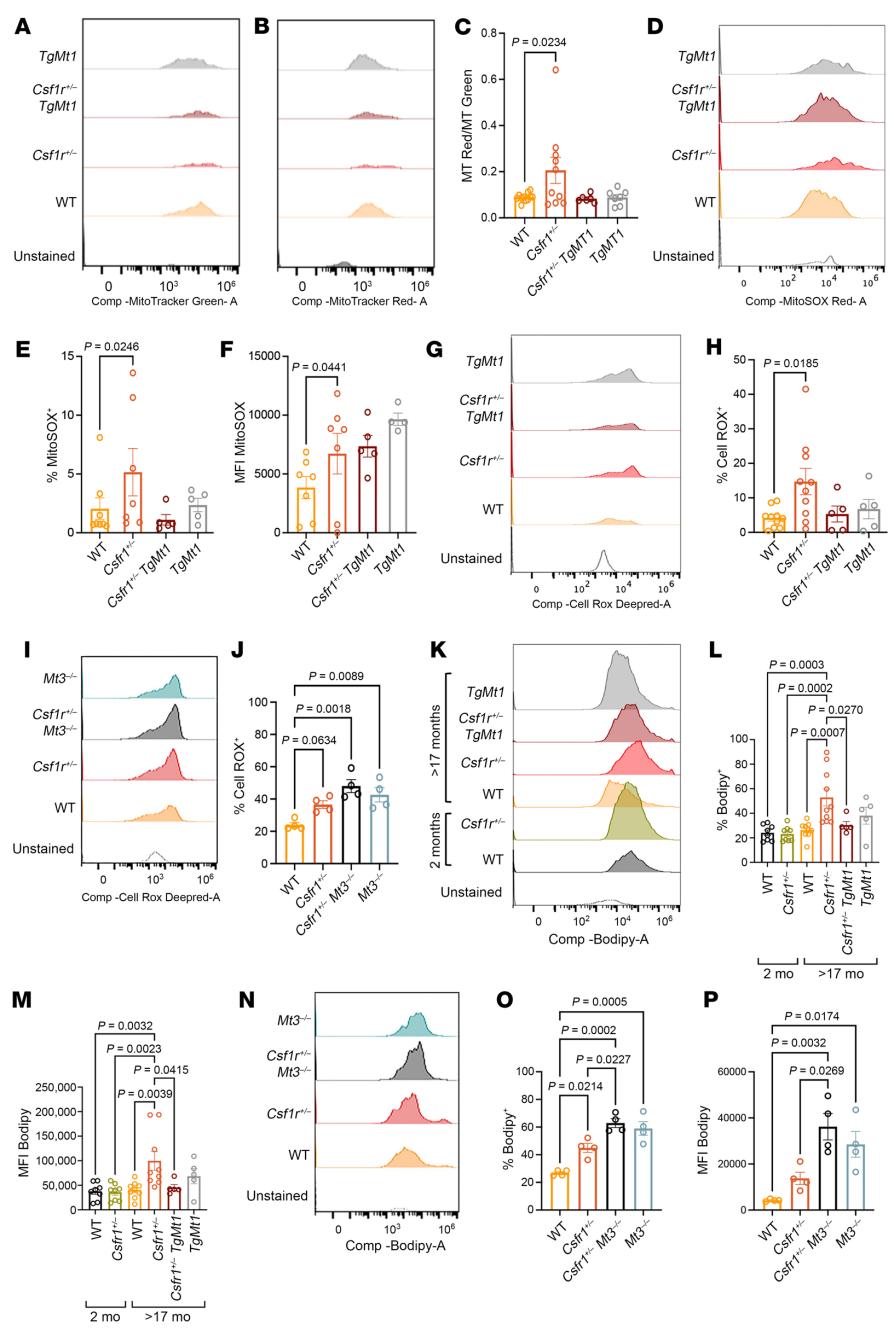
Effects of MT overexpression and deficiency on mitochondrial activity, ROS production, and on accumulation of lipid droplet–containing macrophages in *Csf1r^+/–^* mice. (**A**–**C**) Assessment of mitochondrial polarization in brain macrophages. (**A** and **B**) Representative histograms of macrophages stained with Mitotracker green (MT Green) to estimate mitochondrial mass and with Mitotracker red (MT Red) to estimate mitochondrial polarization. (**C**) Ratio of median fluorescence intensity (MFI) (MT Red/MT Green). (**D**–**F**) Effects of *Csf1r* heterozygosity and *Mt1* overexpression on mitochondrial superoxide production by macrophages. (**D**) Representative histograms and (**E** and **F**) quantitation. (**G**–**J**) Effects of *Csf1r* heterozygosity, *Mt1* overexpression, and *Mt3* deletion on cellular ROS production by macrophages. (**G** and **I**) Representative histograms and (**H** and **J**) quantitation. (**K**–**P**) Measurement of lipid droplet–containing macrophages. (**K** and **N**) Representative histograms showing the distribution of Bodipy^+^ macrophages relative to the unstained control and to one another. Quantitation of the percentage Bodipy^+^ macrophages (**L** and **O**) and quantitation of Bodipy MFI (**M** and **P**). Each circle represents 1 mouse. The ability of *Mt1* overexpression to protect *Csf1r^+/–^* mice was evaluated at >17 months of age (**A**–**H** and **K**–**M**), while the exacerbation of pathology due to *Mt3* deletion was evaluated at 8–9 months of age (**I**, **J**, and **N**–**P**). Means ± SEM. One-way ANOVA, followed by Dunnett’s (**C**, **E**, **F**, and **H**) or Tukey’s (**J**, **L**, **M**, **O**, and **P**).

MTs have an important and multifaceted antioxidant role. They buffer redox-active Fe^2+^ and Cu^+^ and function as direct scavengers of NO and ROS (28). In addition, through chelation and release of Zn^2+^, they regulate mitochondrial respiration (45), the activity of NADPH oxidase (46), and the production of mitochondrial and cytosolic ROS. We therefore hypothesized that their downregulation in the brains of *Csf1r^+/–^* mice could contribute to oxidative stress and investigated how genetic manipulation of MT activity contributes to the regulation of mitochondrial activity and of ROS production in the brain. To this end, we probed mitochondrial potential using Mitotracker red (MT Red), a fluorescent dye that accumulates in mitochondria in amounts directly proportional to their membrane potential. To account for possible differences in mitochondrial mass, the fluorescence of MT Red was normalized to that of Mitotracker green (MT Green), a fluorescent probe that labels all polarized mitochondria regardless of their variations in membrane potential. As shown in Figure 7, A–C, *Csf1r* heterozygosity selectively increased mitochondrial membrane polarization in CD45^+^ CD11b^+^ brain macrophages while neural lineage and endothelial cells continued to retain normal mitochondrial phenotypes (Supplemental Figure 7). Overexpression of *Mt1* attenuated mitochondrial hyperpolarization (Figure 7, A–C) and reduced mitochondrial superoxide levels (Figure 7, D–F) in macrophages. Furthermore, overexpression of *Mt1* attenuated the increase in total cellular ROS in macrophages (Figure 7, G and H). In contrast, the absence of MT3 exacerbated the increase in cellular ROS in presymptomatic *Csf1r^+/–^* mice (Figure 7, I and J).

### Overexpression of Mt1 reduces the accumulation of lipid droplet–containing macrophages in aged Csf1r^+/–^ mice.

The release of ROS from microglia can affect the neighboring cells, leading to demyelination, one of the main pathological features of late CRL. In the process of clearing myelin and cellular debris, microglia store the excess cholesterol they engulf in lipid droplets (47). In pathological conditions, this leads to the accumulation of lipid-laden macrophages (48). Thus, we reasoned that measurement of lipid droplet accumulation in microglia could offer an indirect measure of pathology. Indeed, at 2 months of age in the absence of demyelination or neuronal cell death (27), there was no difference in the lipid droplet content in brain macrophages of *Csf1r^+/–^* mice compared with WT (Figure 7, K–M). However, by 17 months of age, *Csf1r^+/–^* mice had an increased percentage of lipid droplet–containing macrophages and a higher median lipid droplet load (Figure 7, K–M). Consistent with their improved behavioral results, *Csf1r^+/–^* *TgMt1* mice exhibited a significant reduction in lipid droplet–containing macrophages, indicating an amelioration of pathology (Figure 7, K–M). In contrast, *Mt3* deficiency exacerbated the accumulation of lipid droplets in macrophages and increased the frequency of lipid droplet–containing macrophages in presymptomatic, 8- to 9-month-old *Csf1r^+/–^* mice (Figure 7, N–P).

Together, our data indicate that CSF-1R signaling suppresses ROS production in brain macrophages, as well as the subsequent pathology, through regulation of MT expression and labile Fe^2+^ brain load.

## Discussion

Leukodystrophies are a heterogeneous group of genetic disorders that affect the cerebral white matter. Glial cells are affected, their dysregulation leading to myelin pathology and secondary axonal pathology (49). The pathological mechanisms vary widely and include oxidative stress, metabolic and energy production deficits, as well as disruption of microglial homeostasis. Among the adult-onset leukodystrophies, CRL is the prototypical example of microglial-mediated leukodystrophy (15). However, the primary microglial deficit caused by insufficient CSF-1R signaling is currently unknown. To approach this question, we employed snRNA-seq of brains of young, 2-month-old, *Csf1r^+/–^* mice before the appearance of pathological or behavioral alterations. The 28 clusters resolved were represented equally in cells from *Csf1r^+/–^* and control mice. Significant changes in gene expression between *Csf1r^+/–^* and WT mice were mostly observed in microglia. Cells that do not express the CSF-1R, including oligodendrocytes (cluster 9) and neural lineage (cluster 29) and endothelial (cluster 22) cells were also affected though to a lesser extent. Thus, in young mice, although microglia do not exhibit features associated with inflammatory activation, their altered state affects the status of endothelial cells, oligodendrocytes, and subsets of neurons.

Within *Csf1r^+/–^* microglia of the 2-month-old mice, the expression of genes associated with pathogenic or homeostatic microglial states was not altered. Instead, there was an enrichment in differentially expressed transcripts related to inflammation, energy production, and ion transport and homeostasis. The transcriptomic profiles indicate that reduction of CSF-1R signaling causes alterations of metal ion homeostasis in microglia (Figure 2 and Supplemental Figure 2), with concomitant activation of cell death pathways and suppression of the eIF2 pathway, in neuronal cells and oligodendrocytes (Figure 3).

We showed that *Csf1r^+/–^* brain macrophages accumulated labile Zn^2+^ and Cu^+^ and that the levels of Cu^+^ were reduced in the endothelial cells of young, disease-free *Csf1r^+/–^* mice. The brain is the organ with the second-highest Cu^+^ concentration in the body (50). As a cofactor for multiple enzymes, including superoxide dismutase, Cu plays a key role in maintaining the redox balance of the brain (51). Cu deficiency in endothelial cells can lead to increased levels of superoxide ion and low levels of NO, a crucial vasodilator, negatively influencing blood vessel health and brain function (52). Indeed, structural vascular anomalies (31, 32) and recurrent hypoxic-ischemic injuries have been postulated to contribute to microglial activation and demyelination in CRL (reviewed in ref. 12).

At 2 months of age, the accumulation of labile Zn^2+^ in *Csf1r^+/–^* brain macrophages (Figure 2G) did not correlate with reduced levels in other cell types. However, starting at 8 months of age, a significant decrease in labile Zn^2+^ became evident, initially in endothelial cells (Supplemental Figure 6A) and, by 20 months of age, in neural lineage cells (Figure 6A). Zn^2+^ is essential for brain function (53). Through interaction with myelin structural proteins (e.g., MBP, MAG), Zn^2+^ participates in the stabilization of myelin structure (54, 55). Furthermore, labile Zn^2+^ partially mediates neuronal intracellular signaling and contributes to synaptic transmission in zincergic neurons (56). Therefore, we speculated that chronic retention of Zn^2+^ and Cu^+^ in *Csf1r^+/–^* microglia could result in decreased bioavailability of these trace elements to neural lineage and endothelial cells, leading to chronic stress and ultimately, pathology. Indeed, we observed suppression of the eIF2 pathway in oligodendrocytes and subpopulations of neurons of *Csf1r^+/–^* mice. Inhibition of the eIF2 pathway is a key indicator of activation of the integrated stress response (ISR) (34, 57), and mutations in eIF2B that hypersuppress translation lead to vanishing white matter disease, a demyelinating leukoencephalopathy (58). However, when the Gene CLIC ISR signature (36) was run as a Gene Ontology (GO) term against each of the clusters, we found no evidence for ISR activation (Supplemental Figure 8). Furthermore, while treatment of *Csf1r^+/–^* mice with an eIF2B activator tended to improve motor performance, it was not sufficient to prevent the development of cognitive deficits, suggesting that inhibition of the eIF2 pathway is not a major contributor to CRL.

Relevant to the disruption of metal ion homeostasis, a remarkable feature of the single-cell transcriptomic changes observed in *Csf1r^+/–^* mice was the downregulation of *Mts* *1* and *3*. We found that *Mts* *2* and *3* were downregulated in *Csf1r*-expressing microglia, while *Mt1*, alone or in combination with *Mt3*, was downregulated in oligodendrocytes and neurons that do not express *Csf1r*. *Mt1* was downregulated in 21 of the 28 clusters examined, while *Mt3* was downregulated in 15 clusters, including microglia (cluster 21), oligodendrocytes (cluster 9), and numerous subsets of excitatory and inhibitory neurons. While *Mt1* expression is highly dependent on Zn (59), we did not observe a decrease in labile Zn load in neural lineage cells of young mice. The mechanism contributing to the extensive downregulation of *Mt1* remains to be explored. In contrast, *Mt3* expression is unresponsive to Zn supplementation (60). However, pharmacological inhibition of CSF-1R has been reported to reduce the expression of *Mt3* in microglia (61), suggesting that *Mt3* deficiency in microglia is a consequence of *Csf1r* heterozygosity.

MTs are small, thiol-rich proteins that can bind Zn, Cu, Cd, and, at lysosomal pH, also Fe^2+^ (28). Although they have a higher affinity for Cd and Cu than for Zn, they play a particularly important role in controlling the storage and redistribution of Zn^2+^ (43). MTs move from the cytosol to cellular compartments and are secreted and taken up by cells (43). In this manner, they contribute both to the buffering of Zn^2+^ in the steady state and to the cellular redistribution and compartmentalization of transiently elevated Zn^2+^ concentrations in altered states. Zn^2+^ affects the state and activity of more than 2,000 brain proteins through high-affinity interaction with their catalytic and/or interface sites (62). Thus, minor changes in the availability of cellular zinc ions can elicit significant physiological responses. Relevant to our work, the dynamic control of Zn^2+^ bioavailability by MTs is important in redox homeostasis. In the mitochondrial intermembrane space, the release of Zn^2+^ from MTs was shown to inhibit the activity of the electron transport chain (45), which is a significant source of cellular ROS (63). In contrast, under stress conditions (e.g., hypoxia, oxidative stress, mild acidification, excess Cu or Cd), Zn^2+^ is released from MTs in the cytosol and can contribute to the activation of NADPH oxidase with subsequent production of ROS (46). Indeed, increased levels of Zn^2+^ in microglia trigger an increased production of ROS in an NADPH oxidase–dependent manner (64, 65). The release of ROS from microglia can affect the neighboring cells, particularly oligodendrocytes, which, due to their high iron and low antioxidant levels, are exquisitely sensitive to oxidative stress (66). Furthermore, it has been shown that ROS release by microglia can cause the release of Zn^2+^ from intracellular stores and a K^+^ current surge in neurons, ultimately leading to neuronal cell death (67), which, interestingly, could be mitigated by neuronal overexpression of *Mt3*. We therefore hypothesized that the elevation of Zn^2+^ levels in brain macrophages could induce their production of ROS, which, on the background of extensive downregulation of *Mt1* and *Mt3* expression in oligodendrocytes and neurons, will contribute to disease pathology.

To test our hypothesis, we genetically manipulated MT activity in *Csf1r^+/–^* mice. As expected, the overexpression of *Mt1* compensated for the negative effects of decreased CSF-1R signaling in microglia by reducing the accumulation of Zn^2+^, mitochondrial hyperpolarization, the production of mitochondrial superoxide, and total cellular ROS. Importantly, in aged *Csf1r^+/–^* mice, the overexpression of *Mt1* also reduced the accumulation of labile Fe^2+^ in microglia, oligodendrocytes, and neurons, thereby limiting its participation in Fenton reactions that lead to additional oxidative stress and cellular damage. These actions explain the decreased demyelination and the attenuation of behavioral deficits in *Csf1r^+/^ TgMt1^/+^* compared with *Csf1r^+/–^* mice. Opposite results were observed when *Mt3* was genetically ablated, highlighting the importance of metal ion dyshomeostasis in the development of CRL. Overall, our studies suggest that CSF-1R signaling suppresses both ROS production in brain macrophages and CRL pathology through regulation of MT expression.

The observation that *Csf1r^+/–^* BMM accumulated more Zn, Cu, and Fe than WT counterparts, in the absence of myelin challenge, or pathological stimuli, demonstrates that the CSF-1R directly regulates metal ion levels in macrophages. Thus, the present study identifies what we believe to be a novel biological function of the CSF-1R.

Numerous studies indicate that dysregulation of metal ion homeostasis is an integral component of neurodegenerative and demyelinating diseases. The endogenous copper-binding peptide glycyl-l-histidyl-l-lysine (GHK) has the ability to bind both Cu and Zn ions and attenuate their toxicity in BV2 microglia and primary neuronal cultures in vitro (68) and may be considered as a potential cytoprotective compound for diseases in which Cu and Zn toxicity are involved. However, in diseases involving imbalanced Cu and Zn distribution, such as CRL, where we observe both the accumulation in macrophages and associated deficiency in other cell types, the use of Cu- and Zn-chelating agents may not be appropriate. On the other hand, iron chelation results in an improvement in outcome in Alzheimer’s and Parkinson’s diseases (69–72). Here we show that *Csf1r* haploinsufficiency leads to progressive accumulation of redox-active Fe^2+^ in microglia, followed by its increase in neural lineage and endothelial cells. Furthermore, overexpression of *Mt1* reduces the accumulation of labile Fe^2+^ in all these cells and ameliorates neurological dysfunction. Interestingly, we have observed that a high-fat diet decreases *Mt1* and *Mt3* expression in mouse brain (Supplemental Figure 9) and accelerates mouse CRL development (5). These findings indicate that increased MT activity could suppress CRL development. Zinc supplementation is known to increase MT1 expression. However, its therapeutic effects in CRL are unpredictable, given the early elevation of labile Zn^2+^ in *Csf1r^+/–^* microglia. Caloric restriction is an alternative strategy to increase MT expression in the brain (73). Therefore, iron chelation and caloric restriction may be considered as possible early intervention modalities for CRL.

## Methods

### Sex as a biological variable

The in vivo and ex vivo animal studies included both female and male mice. As no discernable sex-related difference was observed in the evolution of disease, sex was not considered a biological variable. The number, sex, and age of mice used in the experiments are reported in each figure and summarized in Supplemental Table 6. Similarly, since CRL affects both male and female patients, sex was not considered a biological variable for human studies.

### Animal studies

#### Mouse strains, breeding, and maintenance.

*Csf1r^+/–^* mice were generated, maintained, and genotyped as described previously (3, 74). *Mt1*-transgenic mice [B6.Cg-Tg(Mt1)174Bri/J] (40) were purchased from The Jackson Laboratory (stock no. 002210) and genotyped using the provided protocol. *Mt3*-deficient mice (C57BL/6, *Mt3* deletion of exon 3) were a gift from University of Cincinnati, Cincinnati, Ohio, USA, and genotyped as described (42). Cohorts were developed from the progeny of matings of *Csf1r^+/–^* to *WT* mice and of *TgMt1* and *Mt3*^–/–^ mice with *Csf1r^+/–^* mice, randomized with respect to the litter of origin. In some experiments (Supplemental Figure 7), WT C57BL/6 male mice were maintained on the D124921 60% fat diet (Research Diets, Inc.) for a month starting from 2.5 months of age. At 3.5 months, they were weighed and sacrificed to examine the expression of Mts in the cerebral white matter.

### Human studies

Frozen brain tissue blocks containing periventricular white matter were obtained from the Mayo Clinic Brain Bank. Consent for autopsy was obtained from the legal next-of-kin. Information on the CRL patients harboring *CSF1R* mutations and control cases included in this study is summarized in Supplemental Table 7. Frozen brain sections were prepared at Mayo Clinic as described (16). Regions of interest were dissected from the frozen slabs and placed in microcentrifuge tubes before being shipped to the research laboratory on dry ice. At all steps, the fresh and frozen tissue was handled with Universal Precautions.

### Isolation of nuclei from mouse brain

Brain cell nuclei were isolated from 2-month-old WT and *Csf1r^+/–^* mice using a detergent-free nuclei isolation kit (Invent Biotechnologies). Briefly, mice were anesthetized and perfused with 50 mL of ice-cold, RNase-free PBS containing 50 U/mL heparin. The nuclei were separated according to the manufacturer’s instructions. Myelin debris was removed using myelin depletion immunomagnetic beads (Miltenyi Biotec). After the final centrifugation (500*g*, 5 min, 4°C), nuclei were resuspended in 0.5 mL nuclei suspension buffer (2% BSA and 0.2 U/μL RNase inhibitor in RNase-free molecular biology-grade PBS) and filtered through a 40 μm FlowMi Cell Strainer.

### snRNA-seq

Isolated mouse nuclei were subjected to droplet-based, 3′ end, massively parallel snRNA-seq using Chromium Next GEM Single Cell 3′ Kit v3.1 per the manufacturer’s instructions (10x Genomics). The libraries were sequenced using a HiSeq 2500 sequencer (Illumina). Sample demultiplexing, barcode processing, and single-cell transcript counting were performed using Cell Ranger (10x Genomics; version 5.0) (75). We obtained 16,415, 16,301, and 13,409 nuclei for the 3 WT samples, with mean reads per cell of 19,052, 19,174 and 11,974. For the 3 *Csf1r^+/–^* samples, we obtained 9,905, 11,369, and 10,224 nuclei, with mean reads per cell of 29,313, 26,086, and 22,013.

#### snRNA-seq data analysis.

The snRNA-seq data were analyzed by our recently developed scDAPP pipeline (24), which performed cell filtering and other quality controls semiautomatically on each sample and then integrated all samples with the RISC software (v1.7) for clustering (75). In each sample, nuclei with unique molecular identifiers between 500 and 50,000, >200 detected genes, and <5% mitochondrial reads were kept. After this filtering, data from all 6 samples were integrated by the Reference Principal Component Integration method in the RISC package and then clustered with Louvain algorithm using 30 principal components, resolution of 1, and otherwise default parameters. Cluster markers were computed by Wilcoxon’s rank test. Differential expression analysis between WT and *Csf1r^+/–^* cells (for each cluster) was performed using the pseudobulk_mode and the EdgeR-LRT method in scDAPP; i.e., cells from the same mouse were combined and used for statistical testing by edgeR. Genes reaching *P* < 0.01 and expressed in at least 10% of the WT or *Csf1r^+/–^* cells were considered to change significantly. We did not further apply multiple-testing correction because it would yield too few genes for downstream pathway enrichment analysis.

### Proteomic analysis of brain macrophages and oligodendrocytes

Brains were dissociated into single-cell suspensions using an Adult Brain Dissociation Kit (Miltenyi Biotec). Brain macrophages and oligodendrocytes were isolated by immunomagnetic separation, using CD11b and Anti-O4 microbeads (Miltenyi Biotec), respectively, and stored at –80°C. Although throughout the manuscript we use the term “brain macrophages,” the vast majority of these CD11b-positive cells are microglia. For proteomic analysis, cells were lysed in 5% SDS-containing sample buffer, and the proteins were isolated using S-TRAP micro spin columns (Protifi) and digested to obtain peptides for mass spectrometric analysis as described (22, 76). Peptides were desalted using a 96-well plate filter (Orochem) packed with 1 mg of Oasis HLB C-18 resin (Waters). Samples were loaded onto a Dionex RSLC Ultimate 300 (Thermo Fisher Scientific), coupled online with an Orbitrap Exploris 480 (Thermo Fisher Scientific). A 2-column system, consisting of a C-18 trap cartridge (300 μm ID, 5 mm length) and a picofrit analytical column (75 μm ID, 25 cm length) packed in-house with reversed-phase Repro-Sil Pur C18-AQ 3 μm resin, was used for chromatographic separation. Peptides were separated using a 120-minute gradient from 4% to 30% buffer B (buffer A: 0.1% formic acid, buffer B: 80% acetonitrile + 0.1% formic acid) at a flow rate of 300 nL/min. Mass spectrometric data were acquired in a data-dependent acquisition mode. The full MS scan was set to 300–1,200 *m/z* in the Orbitrap with a resolution of 120,000 (at 200 *m/z*) and an automatic gain control (AGC) target of 5 × 10^5^. MS/MS was performed in the ion trap using the top speed mode (2 s), an AGC target of 1 × 10^4^, and an higher-energy collisional dissociation collision energy of 35. Proteome raw files were searched using the Proteome Discoverer software (v2.4, Thermo Fisher Scientific) using the SEQUEST search engine and the SwissProt mouse database. Variable modification of N-terminal acetylation and fixed modification of carbamidomethyl cysteine were included in the search. Trypsin was specified as the digestive enzyme with up to 2 missed cleavages allowed. Mass tolerance was set to 10 ppm for precursor ions and to 0.2 Da for product ions. Protein and peptide FDRs were set to 1%. The data were analyzed as described (76).

### Pathway analysis of the significantly differentially expressed genes

The impact of the significant changes in gene expression or protein abundance detected in each comparison was analyzed using IPA software (QIAGEN).

### Behavioral studies

The behavioral studies were carried out as described (16, 18, 41). Detailed protocols are provided in the Supplemental Methods.

### qRT-PCR

RNA was extracted from the anterior motor cortex and corpus callosum of 3-month-old mice or from human periventricular white matter as described (16, 74). The cDNA was prepared using a Super Script III First Strand Synthesis kit (Invitrogen). Quantitative PCR was performed using SYBR Green in an Eppendorf Realplex II thermocycler. The primers used are listed in Supplemental Table 8.

### Flow cytometric analysis

Single-cell suspensions of brains were obtained as described (77). The cells were stained using cell type–specific antibodies and various combinations of metal (Zinpyr-1, CuCF4, Ferro Orange), mitochondrial (Mito Tracker Green, Mito Tracker Red, MitoSOX Red), and lipid droplet (Bodipy 403/503) dyes as described in the Supplemental Methods. Samples were analyzed in an Aurora CS spectral flow cytometer (Cytek Biosciences). The antibodies and dyes used for staining are listed in Supplemental Tables 9 and 10, and the gating strategy utilized to identify each cell type is shown in Supplemental Figure 10. Data were analyzed using FlowJo.

### Spectromicroscopy of BMM

BMM were prepared from femur flushed bone marrow as previously described (78). Aliquots of cell suspension (10^4^ cells/5 μL) were deposited on Si_3_N_4_ substrate frames (5,000 × 5,000 × 525 μm) with membrane size of 1,000 × 1,000 × 1 μm (Silson Ltd) and air-dried at room temperature overnight in a fume hood. Dried samples were stored at –80°C in 200 μL capped tubes before microscopic examination. OPTIR measurements were performed using the bench-top mIRage photothermal IR instrument (Photothermal Spectroscopy Corp.) located at the SMIS beamline at the SOLEIL Synchrotron.

Synchrotron Radiation X Ray Fluorescence microscopy was carried out at NanoMAX, a hard x-ray nanoprobe beamline at the 3 GeV storage ring at the MAX IV Synchrotron radiation facility in Lund, Sweden. Additional information regarding sample preparation and analysis is provided in the Supplemental Methods.

### Statistics

Statistical analysis was conducted using the GraphPad Prism 8 software. Data were analyzed for the identification of outliers using the Grubbs’ method and for Gaussian distribution by the Shapiro-Wilk normality test. The screened data were further analyzed using 1-tailed Student’s *t* test, or by 1- or 2-way ANOVA, as indicated in the figure legends. Differences between genotypes were analyzed by post hoc multiple-comparison tests (Dunnett, Tukey, and Bonferroni, as indicated in each figure). The level of significance was set at *P* < 0.05. Data within each group are presented as mean ± SEM.

### Study approval

All mouse in vivo experiments were conducted in accordance with the *Guide for the Care and Use of Laboratory Animals* (National Academies Press, 2011) and approved by the Albert Einstein College of Medicine Institutional Animal Care and Use Committee. Studies involving autopsy tissue are exempt from human subject research (Health and Human Services Regulation 45 CFR Part 46).

### Data availability

snRNA-seq data generated during this study have been deposited at NCBI GEO with the project accession number GSE305930 and are publicly available as of the date of publication. Proteomic data generated during this study are provided in Supplemental Tables 4 and 5. The proteomics raw files have been uploaded to the ProteomeXchange Consortium via the PRIDE partner repository with the project accession number PXD067561. Numeric data used to generate the charts are listed in the Supporting Data Values table. Any additional information required to reanalyze the data reported in this paper is available from the corresponding author upon request.

## Author contributions

VC and ERS designed the study and wrote the manuscript. VC, DR, YL, WC, and DZ performed the single-nucleus transcriptomic analyses; JTA and SS the proteomic analyses; VC the histology; VC and DS the flow cytometry studies; and VC, VDK, and AS the OPTIR and XRF studies. PU, IANS, and CS contributed to the OPTIR experiments and PU, IANS, OK, and UJ to the XRF measurements. JA and ERS managed the mouse colony, and JA performed the behavioral experiments. KSV provided the *Mt3^–/–^* mice. ZKW and DWD provided the postmortem brain tissue, and VC performed human gene expression analysis. All authors reviewed and edited the manuscript and approved the final version to be submitted.

## Conflict of interest

ZKW serves as principal investigator (PI) or co-PI on Biohaven Pharmaceuticals Inc. (BHV4157-206) and ONO-2808-03 projects/grants. ZKW also serves as co-PI of the Mayo Clinic APDA Center for Advanced Research, as an external advisory board member for Savanna Biotherapeutics Inc., and as a consultant for Eli Lilly & Company.

## Funding support

This work is the result of NIH funding, in whole or in part, and is subject to the NIH Public Access Policy. Through acceptance of this federal funding, the NIH has been given a right to make the work publicly available in PubMed Central.

- NIH grant R01NS091519 (ERS).
- David and Ruth Levine funding (ERS).
- NIH P50 HD105352 (Rose F. Kennedy IDDRC).
- Hevolution Foundation (AFAR), Einstein-Mount Sinai Diabetes Center, and NIH Office of the Director S10OD030286 (Sidoli lab).
- Crafoord Foundation 20191011 (PU).
- Carl Trygger Foundation CTS 22:2138 (AS).
- Hjärnfonden FO2025-0263-HK-189 (OK).
- JPND 2024-02266 (OK).
- Olle Engkvists Stiftelse 220-0182 (OK).
- Vetenskapsrådet Swedish Research Council VR 2018-07152, Vinnova (Swedish Governmental Agency for Innovation Systems) 2018-04969, Formas 2019-02496 (MAX IV, a Swedish national user facility).
- NIH/NIA and NIH/NINDS 1U19AG063911 (ZKW).
- Haworth Family Professorship in Neurodegenerative Diseases fund (ZKW).
- Albertson Parkinson’s Research Foundation (ZKW).
- PPND Family Foundation (ZKW).

## Supplementary Material

Supplemental data

Supplemental table 1

Supplemental table 2

Supplemental table 3

Supplemental table 4

Supplemental table 5

Supporting data values

## Version 1 02/26/2026

In-Press Preview

## Version 2 05/01/2026

Electronic publication

## References

[1] Stanley ER, Chitu V (2014). CSF-1 receptor signaling in myeloid cells. Cold Spring Harb Perspect Biol.

[2] Boyce BF (2013). Advances in the regulation of osteoclasts and osteoclast functions. J Dent Res.

[3] Dai XM (2002). Targeted disruption of the mouse colony-stimulating factor 1 receptor gene results in osteopetrosis, mononuclear phagocyte deficiency, increased primitive progenitor cell frequencies, and reproductive defects. Blood.

[4] Dai XM (2004). Osteoclast deficiency results in disorganized matrix, reduced mineralization, and abnormal osteoblast behavior in developing bone. J Bone Miner Res.

[5] Chitu V (2022). Modeling CSF-1 receptor deficiency diseases - how close are we?. FEBS J.

[6] Rademakers R (2011). Mutations in the colony stimulating factor 1 receptor (CSF1R) gene cause hereditary diffuse leukoencephalopathy with spheroids. Nat Genet.

[7] Konno T (2014). Haploinsufficiency of CSF-1R and clinicopathologic characterization in patients with HDLS. Neurology.

[8] Konno T (2017). Clinical and genetic characterization of adult-onset leukoencephalopathy with axonal spheroids and pigmented glia associated with CSF1R mutation. Eur J Neurol.

[9] Konno T (2018). *CSF1R*-related leukoencephalopathy: a major player in primary microgliopathies. Neurology.

[10] Ali ZS (2007). A comparative morphologic analysis of adult onset leukodystrophy with neuroaxonal spheroids and pigmented glia--a role for oxidative damage. J Neuropathol Exp Neurol.

[11] Barsukova AG (2012). Focal increases of axoplasmic Ca2+, aggregation of sodium-calcium exchanger, N-type Ca2+ channel, and actin define the sites of spheroids in axons undergoing oxidative stress. J Neurosci.

[12] Chitu V (2021). Colony stimulating factors in the nervous system. Semin Immunol.

[13] Ginhoux F (2010). Fate mapping analysis reveals that adult microglia derive from primitive macrophages. Science.

[14] Nandi S (2012). The CSF-1 receptor ligands IL-34 and CSF-1 exhibit distinct developmental brain expression patterns and regulate neural progenitor cell maintenance and maturation. Dev Biol.

[15] Biundo F (2021). Microglial reduction of colony stimulating factor-1 receptor expression is sufficient to confer adult onset leukodystrophy. Glia.

[16] Chitu V (2020). Microglial homeostasis requires balanced CSF-1/CSF-2 receptor signaling. Cell Rep.

[17] Arreola MA (2021). Microglial dyshomeostasis drives perineuronal net and synaptic loss in a CSF1R^+/-^ mouse model of ALSP, which can be rescued via CSF1R inhibitors. Sci Adv.

[18] Biundo F (2023). Elevated granulocyte colony stimulating factor (CSF) causes cerebellar deficits and anxiety in a model of CSF-1 receptor related leukodystrophy. Glia.

[19] Kempthorne L (2020). Loss of homeostatic microglial phenotype in CSF1R-related Leukoencephalopathy. Acta Neuropathol Commun.

[20] Pan J (2024). Deciphering glial contributions to CSF1R-related disorder via single-nuclear transcriptomic profiling: a case study. Acta Neuropathol Commun.

[21] Du S (2025). Mutations in the human CSF1R gene impact microglia’s maintenance of brain white matter integrity. Nat Immunol.

[22] Chitu V (2023). Prophylactic effect of chronic immunosuppression in a mouse model of CSF-1 receptor-related leukoencephalopathy. Glia.

[23] Li X (2023). Minocycline protects against microgliopathy in a Csf1r haplo-insufficient mouse model of adult-onset leukoencephalopathy with axonal spheroids and pigmented glia (ALSP). J Neuroinflammation.

[24] Ferrena A (2024). scDAPP: a comprehensive single-cell transcriptomics analysis pipeline optimized for cross-group comparison. NAR Genom Bioinform.

[25] Oyanagi K (2017). Adult onset leukoencephalopathy with axonal spheroids and pigmented glia (ALSP) and Nasu-Hakola disease: lesion staging and dynamic changes of axons and microglial subsets. Brain Pathol.

[26] Kinoshita M (2021). Pathologic basis of the preferential thinning of thecorpus callosum in adult-onset leukoencephalopathy with axonal spheroids and pigmented glia (ALSP). eNeurologicalSci.

[27] Biundo F (2023). Trem2 enhances demyelination in the Csf1r^+/-^ mouse model of leukoencephalopathy. Biomedicines.

[28] Pavic M (2019). Forgotten partners and function regulators of inducible metallothioneins. Arh Hig Rada Toksikol.

[29] Das Gupta K (2024). CFTR is required for zinc-mediated antibacterial defense in human macrophages. Proc Natl Acad Sci U S A.

[30] Paulus A (2022). Correlative imaging to resolve molecular structures in individual cells: substrate validation study for super-resolution infrared microspectroscopy. Nanomedicine.

[31] Lin WL (2010). Hereditary diffuse leukoencephalopathy with spheroids: ultrastructural and immunoelectron microscopic studies. Int J Clin Exp Pathol.

[32] Delaney C (2020). Attenuated CSF-1R signalling drives cerebrovascular pathology. EMBO Mol Med.

[33] Llanos S, Serrano M (2010). Depletion of ribosomal protein L37 occurs in response to DNA damage and activates p53 through the L11/MDM2 pathway. Cell Cycle.

[34] Bond S (2020). The integrated stress response and phosphorylated eukaryotic initiation factor 2α in neurodegeneration. J Neuropathol Exp Neurol.

[35] DuRose JB (2009). Phosphorylation of eukaryotic translation initiation factor 2alpha coordinates rRNA transcription and translation inhibition during endoplasmic reticulum stress. Mol Cell Biol.

[36] Wong YL (2019). eIF2B activator prevents neurological defects caused by a chronic integrated stress response. Elife.

[37] Atrian-Blasco E (2017). Mutual interference of Cu and Zn ions in Alzheimer’s disease: perspectives at the molecular level. Dalton Trans.

[38] Baird SK (2006). Metallothionein protects against oxidative stress-induced lysosomal destabilization. Biochem J.

[40] Palmiter RD (1993). Distal regulatory elements from the mouse metallothionein locus stimulate gene expression in transgenic mice. Mol Cell Biol.

[41] Barker GR, Warburton EC (2011). When is the hippocampus involved in recognition memory?. J Neurosci.

[42] Chowdhury D (2019). Metallothionein 3 controls the phenotype and metabolic programming of alternatively activated macrophages. Cell Rep.

[43] Maret W (2011). Redox biochemistry of mammalian metallothioneins. J Biol Inorg Chem.

[44] Scheiber IF (2014). Metabolism and functions of copper in brain. Prog Neurobiol.

[45] Ye B (2001). Zinc metallothionein imported into liver mitochondria modulates respiration. Proc Natl Acad Sci U S A.

[46] Zhao Y (2018). Synergistic interaction between zinc and reactive oxygen species amplifies ischemic brain injury in rats. Stroke.

[47] Haidar M (2022). Targeting lipophagy in macrophages improves repair in multiple sclerosis. Autophagy.

[48] Van Gerpen JA (2008). Insights into the dynamics of hereditary diffuse leukoencephalopathy with axonal spheroids. Neurology.

[49] Kohler W (2018). Adulthood leukodystrophies. Nat Rev Neurol.

[50] Kardos J (2018). Copper signalling: causes and consequences. Cell Commun Signal.

[51] An Y (2022). The role of copper homeostasis in brain disease. Int J Mol Sci.

[52] Yang SJ (2007). Low nitric oxide: a key factor underlying copper-deficiency teratogenicity. Free Radic Biol Med.

[53] Shippy DC (2024). Zinc utilization by microglia in Alzheimer’s disease. J Biol Chem.

[54] Earl C (1988). Zinc ions stabilise the association of basic protein with brain myelin membranes. J Neurochem.

[55] Kursula P (1999). The small myelin-associated glycoprotein is a zinc-binding protein. J Neurochem.

[56] Zhang C (2022). Neuronal signalling of zinc: from detection and modulation to function. Open Biol.

[57] Ryoo HD (2024). The integrated stress response in metabolic adaptation. J Biol Chem.

[58] Moon SL, Parker R (2018). EIF2B2 mutations in vanishing white matter disease hypersuppress translation and delay recovery during the integrated stress response. RNA.

[59] Miles AT (2000). Induction, regulation, degradation, and biological significance of mammalian metallothioneins. Crit Rev Biochem Mol Biol.

[60] Bousleiman J (2017). Function of metallothionein-3 in neuronal cells: do metal ions alter expression levels of MT3?. Int J Mol Sci.

[61] Soto-Diaz K (2021). Treatment with the CSF1R antagonist GW2580, sensitizes microglia to reactive oxygen species. Front Immunol.

[62] Hubner C, Haase H (2021). Interactions of zinc- and redox-signaling pathways. Redox Biol.

[63] Zhang Y, Wong HS (2021). Are mitochondria the main contributor of reactive oxygen species in cells?. J Exp Biol.

[64] Kauppinen TM (2008). Zinc triggers microglial activation. J Neurosci.

[65] Higashi Y (2011). Microglial zinc uptake via zinc transporters induces ATP release and the activation of microglia. Glia.

[66] Thorburne SK, Juurlink BH (1996). Low glutathione and high iron govern the susceptibility of oligodendroglial precursors to oxidative stress. J Neurochem.

[67] Knoch ME (2008). Microglia induce neurotoxicity via intraneuronal Zn(2+) release and a K(+) current surge. Glia.

[68] Min JH (2024). Glycyl-l-histidyl-l-lysine prevents copper- and zinc-induced protein aggregation and central nervous system cell death in vitro. Metallomics.

[69] Bolognin S (2009). Metal ion physiopathology in neurodegenerative disorders. Neuromolecular Med.

[70] Jomova K (2010). Metals, oxidative stress and neurodegenerative disorders. Mol Cell Biochem.

[71] Bjorklund G (2025). Cerebral iron accumulation in multiple sclerosis: pathophysiology and therapeutic implications. Autoimmun Rev.

[72] Chen L (2025). Homeostasis and metabolism of iron and other metal ions in neurodegenerative diseases. Signal Transduct Target Ther.

[73] Swindell WR (2009). Genes and gene expression modules associated with caloric restriction and aging in the laboratory mouse. BMC Genomics.

[74] Chitu V (2015). Phenotypic characterization of a Csf1r haploinsufficient mouse model of adult-onset leukodystrophy with axonal spheroids and pigmented glia (ALSP). Neurobiol Dis.

[75] Liu Y (2021). Robust integration of multiple single-cell RNA sequencing datasets using a single reference space. Nat Biotechnol.

[76] Aguilan JT (2020). Guide for protein fold change and p-value calculation for non-experts in proteomics. Mol Omics.

[77] Legroux L (2015). An optimized method to process mouse CNS to simultaneously analyze neural cells and leukocytes by flow cytometry. J Neurosci Methods.

[78] Rolfe AJ (2017). In vitro phagocytosis of myelin debris by bone marrow-derived macrophages. J Vis Exp.

[79] Dissing-Olesen L (2023). FEAST: a flow cytometry-based toolkit for interrogating microglial engulfment of synaptic and myelin proteins. Nat Commun.

