# Supplementary material for: Disruption of CSF-1 receptor–mediated metal ion homeostasis in the murine brain promotes neurodegenerative disease
Source: J Clin Invest. 2026 Feb 26;136(9):e200121. doi: 10.1172/JCI200121 (PMC13132378; doi:10.1172/JCI200121)
Supplement: Supplemental data [file jci-136-200121-s183.pdf]

Supplementary methods, figures and tables

Disruption of CSF-1 receptor-mediated metal ion homeostasis in the murine brain promotes neurodegenerative disease.

Violeta Chițu[1], Julia Alvarenga[1], Wenna Chen[2], David Reynolds[2], Yang Liu[2], Daqian Sun[3], Anders Sandell[4], Virginjia Danylaité-Karrenbauer[5,6], Per Uvdal[7], Iran A. N. da Silva[8,9,10], Christophe Sandt[11], Oxana Klementieva[8,12], Ulf Johansson[13], Kavitha Subramanian Vignesh[14], Zbigniew K. Wszolek[15], Dennis W. Dickson[16], Jennifer T. Aguilian[17], Simone Sidoli[18], Deyou Zheng[2,19], E. Richard Stanley[1,*]

Supplementary methods:

Flow cytometric analysis

Brains were dissected, minced and digested in 2 mL digestion buffer (2mg/ml Collagenase D, 1U /ml DNase I in Hanks' Balanced Salt solution (HBSS)) for 15 min at 37°C. Myelin was removed by centrifugation in 37% Percoll in HBSS for 10 minutes at 500 x g, without brakes. The cell pellet was washed with Dulbecco's PBS (DPBS), and red blood cells were lysed with ACK buffer. After another wash with DPBS, cells were resuspended in FACS buffer (1% FCS in PBS) at $4x10^5$/ml, divided into aliquots of $4x10^5$ cells for different types of staining and stored on ice. For measurements of intracellular ROS, cells were centrifuged and resuspended in 1ml RPMI with 10%FCS and 500nM Cell ROX Deep Red reagent. Cells were incubated for 30 min at 37°C, washed, resuspended in 100 μL FACS buffer, then subjected to cell surface antigen staining as described below. The Fc receptors were blocked by incubation in FACS buffer containing rat anti-mouse CD16/CD32 (Fc block) for 15 minutes on ice. The cells were stained using antibodies to macrophage (CD45 and CD11b),

oligodendrocyte (O4) and neuronal (CD49a) markers diluted 1:100 in FACS buffer and incubated for 15 min. at room temperature, in the dark. Cells were subsequently washed twice with DPBS and resuspended in 0.5ml DPBS containing various combinations of metal (Zinpyr-1, CuCF4), mitochondrial (Mito Tracker Green, Mito Tracker Red, MitoSOX Red) and lipid droplet (Bodipy 403/503) dyes. To measure labile iron, the samples stained with Zinpyr-1 were washed and the cells resuspended in 0.5ml HBSS containing 1μM Ferro Orange and incubated for 15 min at 37°C. Samples were analyzed in an Aurora CS spectral flow cytometer (Cytek Biosciences). The antibodies and dyes used for staining are listed in Supplemental Tables 9 and 10 and the gating strategy utilized to identify each cell type is shown in Supplemental Figure S10. Data were analyzed using FlowJo.

Spectromicroscopy of bone marrow-derived macrophages

Preparation of macrophages

Bone marrow-derived macrophages were prepared from femur flushed bone marrow as previously described (1). Macrophages were propagated in α-MEM containing 10%FCS and 140ng/ml human recombinant CSF-1. After 23 days in culture, the medium was removed and the cell monolayers were rinsed twice with sterile PBS. Cells were harvested in PBS using a sterile plastic cell lifter (Fisher Scientific) counted, centrifuged (5 min, 4°C, 400xg) and resuspended at 10^6 cells/ml in PBS with 4% PFA. Cells were fixed by incubation at room temperature for 15 min, centrifuged (5 min, 1,000xg) and washed twice with deionized water to remove contaminating substances, then resuspended at 10^7/ml in deionized water. Aliquots of 5μl cell suspension were deposited on Si_3N_4 substrate frames (5000x5000x525μm) with membrane size of 1000x1000x1μm (Silson Ltd) and air dried at room temperature overnight in a fume hood. A minimum of 3 replicates was prepared for each mouse. Dried samples were stored at -80°C in 200μl capped tubes before microscopic examination.

Optical Photothermal Infrared Spectroscopy (O-PTIR) measurements

O-PTIR measurements were performed using the bench-top mIRage photothermal IR instrument (Photothermal Spectroscopy Corp., USA) located at the SMIS beamline at the SOLEIL synchrotron. The instrument features a MIRCat tunable pulsed Quantum Cascade IR Laser (Daylight Solutions, San Diego, CA, USA), scanning 920 to 1800 and 2700 to 3026 cm^{-1} at a pulse rate of 100 kHz. The probe laser was a Continuous Wave Cobolt 532 nm laser and its photothermally-induced modulation was recorded with an Avalanche Photo Diode detector (APD). Hyperspectra of individual cells with 2 cm^{-1} spectral resolution and 400 nm spatial resolution were recorded in reflection mode using a Schwarzschild 40×/0.78 objective (Pike Technologies, Madison, WI, USA). Maps highlighting lipids were extracted by choosing a window between 2840 and 2970 cm^{-1}.

Synchrotron Radiation X Ray Fluorescence (SR-XRF) microscopy

XRF enables label-free, picomolar-sensitive mapping of elemental distributions in biological samples at nanoscale resolution. The XRF microscopy measurements were carried out at NanoMAX, a hard X-ray nanoprobe beamline at the 3 GeV storage ring at the MAX IV synchrotron radiation facility in Lund, Sweden. The beamline utilizes the uniquely high brilliance to achieve nanometer sized coherent focus with high photon intensity, as described in previous work (2). The work in this study was performed at the diffraction station at the beamline (3). The 2D X-ray fluorescence microscopy images were acquired at 12 keV photon energy using a RaySpec single-element silicon drift detector (SDD). The focal spot was 68 × 71 nm (v × h). Images were measured either in 100 nm or 200 nm steps, with the sample placed slightly out of focus to match the step size. The XRF data were normalized to the incident photon intensity by use of an air-filled ion chamber. Fitted elemental 2D maps and associated XRF spectra were extracted using the PyMCA software (4). Since the as-measured XRF images represent 2D projections of the elemental abundance an assessment

emphasizing relative differences associated with enrichment within storage compartments requires a normalization in order to reduce the effect of varying cell thickness. For an internal normalization sulfur is a good measure of the amount of biomass (5). Background subtracted and S-normalized results were obtained using the Fiji (ImageJ) software (6). The box plot shown in Figure 2L is based on S-normalized measurements of 121 cells at 200 nm resolution, comprising 46 cells from three different control wt mice and 72 cells from five different *Csf1r*$^{+/-}$ mice. Emphasis in the analysis was put on well separated cells, excluding clusters. The S-normalized results are qualitatively in very good agreement with absolute concentration measurements using an XRF reference sample (AXO Dresden GMBH) without any correction for cell thickness. In addition, two wt and two *Csf1r*$^{+/-}$ cells, previously imaged with O-PTIR at the SMIS beamline at SOLEIL, were measured at 100 nm resolution.

Behavioral Studies

The behavioral studies were carried out by blinded experimenters and were conducted during the light cycle. To minimize the confounding effect of other factors, the study was randomized and balanced for time (morning versus afternoon), and the two sexes were tested separately.

Cognitive Assessment

Mice were tested for spatial short-term memory in the two-trial test version of the Y-maze (7). The number of entries into the arms and time spent exploring each arm was recorded by the ANY-maze video tracking system (ANY-maze, Stoelting). If spatial memory is intact, the mice are expected to preferentially explore the novel arm. The total number of entries was analyzed to document differences in exploratory activity.

In some experiments, the object placement test was used to gain additional information regarding spatial recognition memory. Each mouse was exposed for 7 min to two identical objects placed in a 40 cm x 40 cm open field box. Four different visual cues were hung on the walls of the box to permit each mouse to orient within the arena. After an interval of 25 minutes, one of the objects (“displaced”) was displaced into a novel position (15 cm distance, 90-degree angle) and each mouse was returned to the same box to explore the objects for 5 min (testing). Time spent exploring each object was recorded.

Motor coordination

Motor coordination was assessed using the balance beam test. In brief, each mouse was placed at one end of a wooden beam (1.6 cm in diameter, 1 m long) elevated 50 cm above the floor and allowed to reach the other end of the beam. The number of slips made while crossing the beam was recorded by the experimenter and used as a measure of locomotor coordination.

Brain connectivity

Brain connectivity was assessed using the temporal order task (8). This task comprised two sample phases. In the first sample phase, the mice were allowed to explore two copies of an identical object for 4 min. After a 1h delay, in sample phase 2, they explored two copies of an object different from the one used in phase 1 for 4 min. In the test trial (3 min duration), given 3 h after sample phase 2, the mice were allowed to explore a copy of the objects from sample phase 1 and a copy of the objects from sample phase for 3 min. If temporal order memory is intact, the mice should spend more time exploring the object from sample 1 presented less recently (the “older” object) compared with the object from sample 2 (the “recent” object). A schematic of the test is shown in Fig. 4B, left panel. Discrimination between the objects was calculated using a weighted difference ratio, calculated as

the absolute difference in the time spent exploring the older and recent objects divided by the total time spent exploring the objects.

References:

1. Rolfe AJ, Bosco DB, Broussard EN, and Ren Y. In Vitro Phagocytosis of Myelin Debris by Bone Marrow-Derived Macrophages. *J Vis Exp.* 2017(130).
2. Johansson U, Carbone D, Kalbfleisch S, Bjorling A, Kahnt M, Sala S, et al. NanoMAX: the hard X-ray nanoprobe beamline at the MAX IV Laboratory. *J Synchrotron Radiat.* 2021;28(Pt 6):1935–47.
3. Carbone D, Kalbfleisch S, Johansson U, Bjorling A, Kahnt M, Sala S, et al. Design and performance of a dedicated coherent X-ray scanning diffraction instrument at beamline NanoMAX of MAX IV. *J Synchrotron Radiat.* 2022;29(Pt 3):876–87.
4. Solé VA, Papillon E, Cotte M, Walter P, and Susini J. A multiplatform code for the analysis of energy-dispersive X-ray fluorescence spectra. *Spectrochimica Acta Part B: Atomic Spectroscopy.* 2007;62:63–8.
5. Schmollinger S, Chen S, Strenkert D, Hui C, Ralle M, and Merchant SS. Single-cell visualization and quantification of trace metals in Chlamydomonas lysosome-related organelles. *Proc Natl Acad Sci U S A.* 2021;118(16).
6. Schindelin J, Arganda-Carreras I, Frise E, Kaynig V, Longair M, Pietzsch T, et al. Fiji: an open-source platform for biological-image analysis. *Nat Methods.* 2012;9(7):676–82.
7. Chitu V, Biundo F, Shlager GGL, Park ES, Wang P, Gulinello ME, et al. Microglial Homeostasis Requires Balanced CSF-1/CSF-2 Receptor Signaling. *Cell Rep.* 2020;30(9):3004–19 e5.
8. Barker GR, and Warburton EC. When is the hippocampus involved in recognition memory? *J Neurosci.* 2011;31(29):10721–31.

Supplementary figures

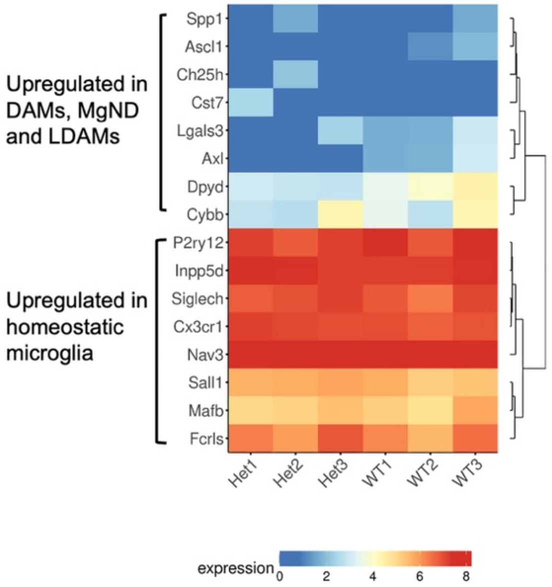

Figure S1. **Expression of transcripts that are typically enriched in neurodegenerative or homeostatic microglial states in Cluster 21 microglia of wt *Csf1r*$^{+/-}$ mice at 2 months of age.** DAM, damage-associated microglia; MGnD, neurodegenerative microglia; LDAM, lipid-droplet-accumulating microglia.

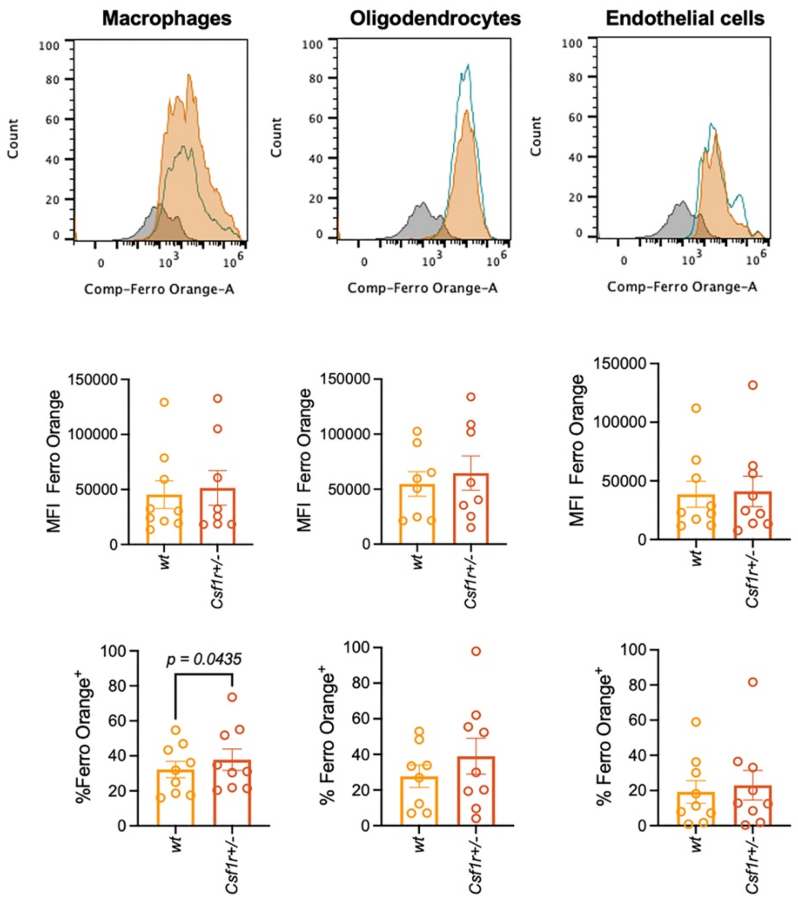

Figure S2. **Changes in the accumulation of labile Fe^{2+} in brain macrophages of young 2-month-old *Csf1r*$^{+/-}$ mice.** Upper panels, representative histograms. Filled gray curves, unstained control; green lined unfilled curve, wt; filled orange curves, *Csf1r*$^{+/-}$. Middle panels, median fluorescence intensity (MFI); lower panels, percent positive cells. Each circle represents one mouse. Means ± SEM. Significance was tested using the one-tailed paired Student's t test.

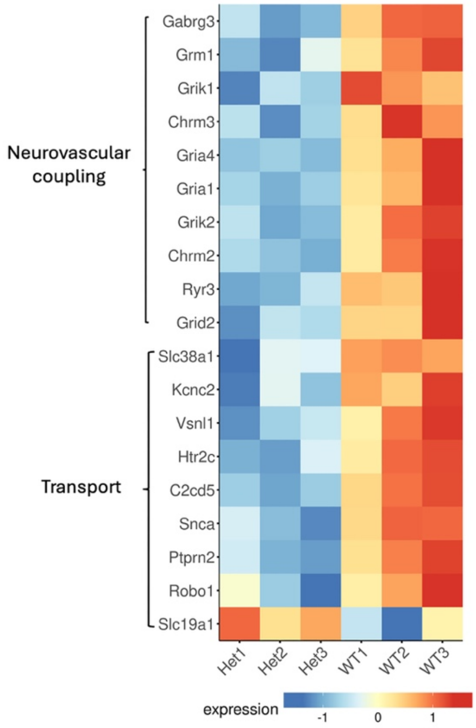

Figure S3. Expression of transcripts associated with neurovascular coupling and endothelial transport in endothelial cell Cluster 22 of 2-month-old wt and *Csf1r*$^{+/-}$ mice.

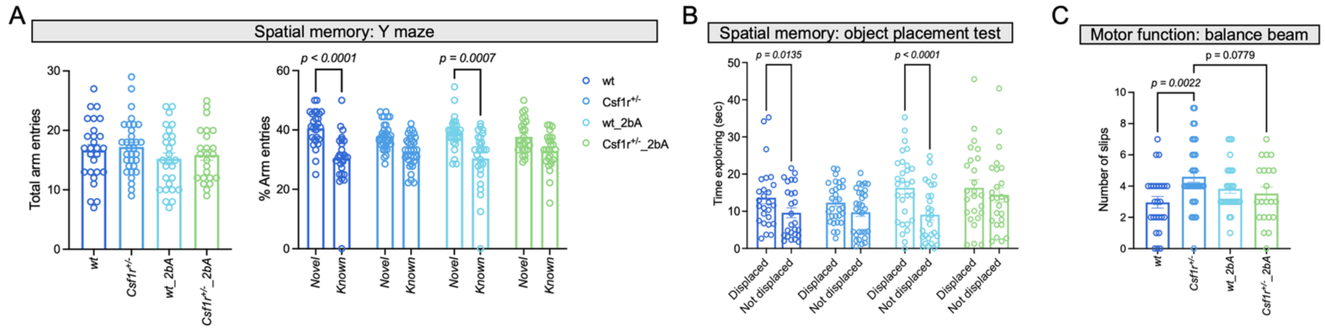

Figure S4. **Minimal benefit from mitigation of the ISR in aged *Csf1r*$^{+/-}$ mice.** Mice were placed on a 2BAct or control diet from 2 months of age. Behavioral characterization was initiated at 16 months of age. **(A, B)** Administration of 2BAct fails to rescue the spatial memory deficits of *Csf1r*$^{+/-}$ mice. (**A**) **Y** maze test. Left panel: There was no change in total exploratory activity among groups (ANOVA p=0.43). Right panel: Spatial memory test. Two- way followed by Bonferroni post-hoc test. (**B**) Object placement test. Two-way ANOVA followed by Bonferroni post-hoc test. (**C**) Administration of 2BAct tends to attenuate the development of motor deficits in *Csf1r*$^{+/-}$ mice. One-way ANOVA, followed by Bonferroni post-hoc test. Each circle represents one mouse. Means ± SEM.

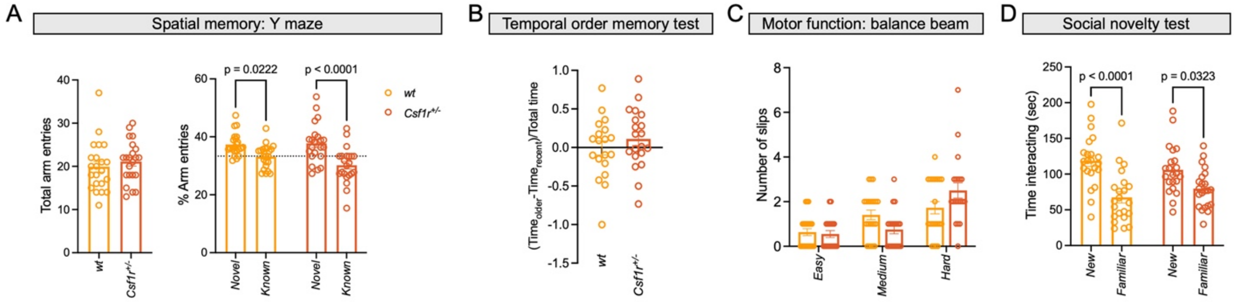

Figure S5. **Absence of behavioral deficits in 7-month-old *Csf1r*$^{+/-}$ mice.** (**A**) Y maze test of spatial memory. *Csf1r*$^{+/-}$mice exhibit no change in exploratory activity (left panel, p=0.24, Student's t test). Right panel, spatial memory is not impaired at this age. Two- way ANOVA, Bonferroni. (**B**) Temporal order memory test. Student's t test p= 0.34.(**C**) Balance beam test indicating absence of motor deficits. Two- way ANOVA, Bonferroni. The labels on the abscissa indicate the difficulty of the test which was inversely proportional to the beam diameter (Easy, 2.25cm; Medium, 1.9cm; Hard, 1.6cm). (**D**) Social novelty test. Two- way ANOVA, Bonferroni. Means ± SEM. Each symbol on the chart represents one mouse.

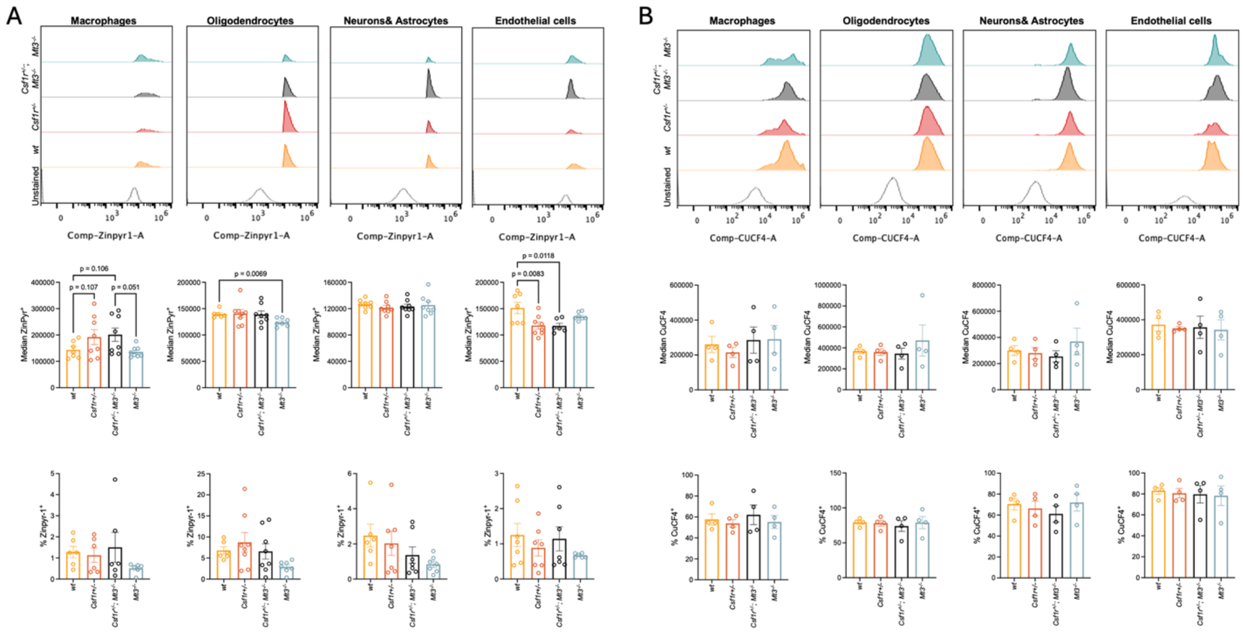

Figure S6. ***Mt3* deletion has no effect on Zn^{2+} or Cu^{+} homeostasis in 8-9-month-old presymptomatic *Csf1r*$^{+/-}$ mice.** (**A**) Levels of labile Zn^{2+} in brain macrophages, neural lineage and endothelial cells. One-way ANOVA followed by Holm-Sidak's post-hoc test. (**B**) Levels of labile Cu^{+}. Upper panels, representative histograms; middle panels, median fluorescence intensity (MFI); lower panels, percent positive cells. Each circle represents one mouse. Means ± SEM.

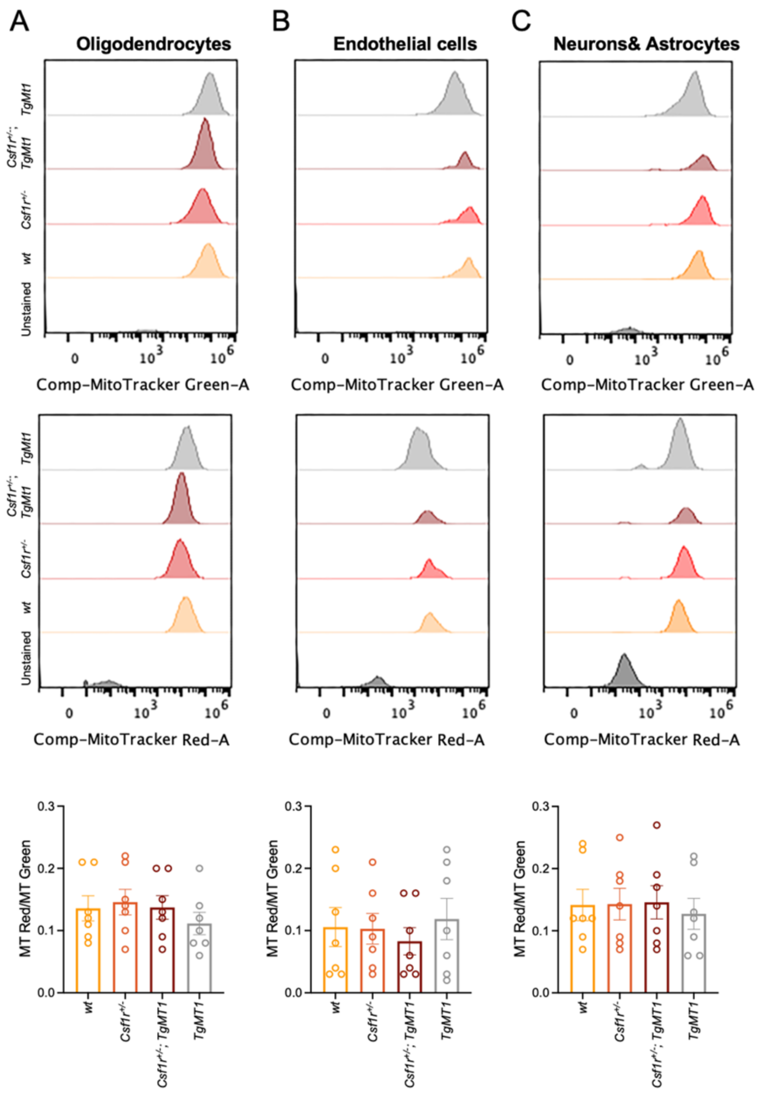

Figure S7. **Normal mitochondrial polarization in neural lineage and endothelial cells of *Csf1r*$^{+/-}$ mice.** (**A-C**) Assessment of mitochondrial polarization in oligodendrocytes, endothelial cells and neurons. Top panels, representative histograms of cells stained with MT Green to estimate mitochondrial mass. Middle panels, representative histograms of cells stained with MT Red to estimate mitochondrial polarization. Lower panels, ratio of median fluorescence intensity (MT Red/MT Green). Each circle represents one mouse. Means ± SEM. Significance was tested using one-way ANOVA.

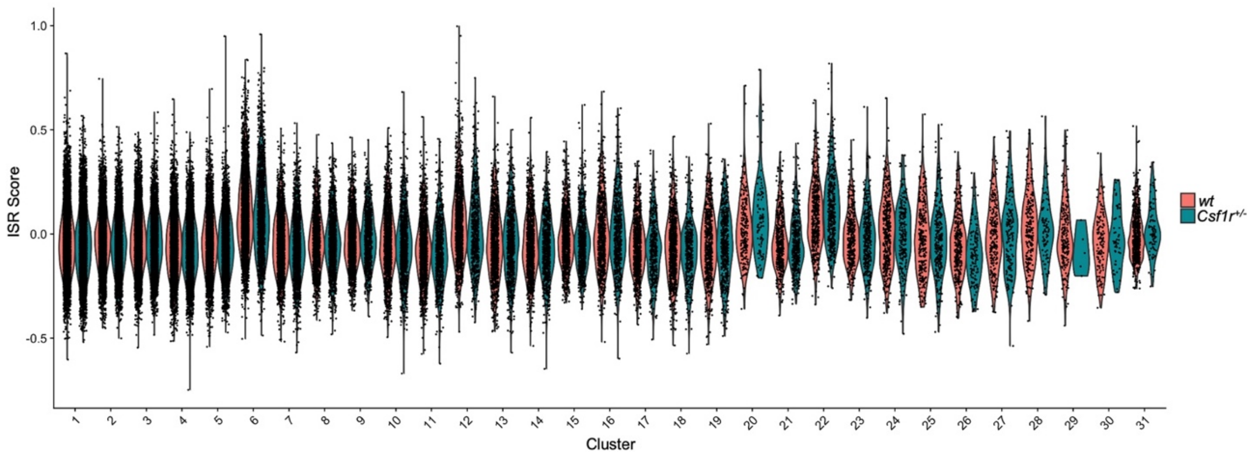

Figure S8. At two months of age, *Csf1r*$^{+/-}$ mice do not exhibit a significant increase in ISR signature gene expression in brain cells compared to wt. Violin plot displaying the ISR activation scores in wt and *Csf1r*$^{+/-}$ cells in each cluster. The width of the violin depicts the density of nuclei at each expression level. Each dot within the violins represents a nucleus.

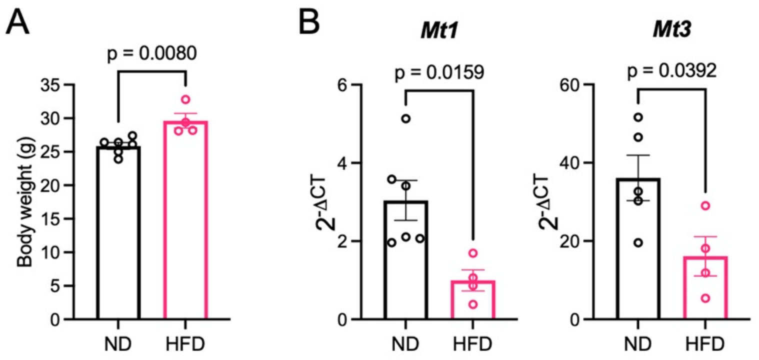

Figure S9. **High-fat diet suppresses the expression of metallothioneins in the brain.** (**A**) Male C57/BL6 mice were fed a high fat diet (HFD) from 2.5 to 3.5 months of age leading to a significant increase in body weight compared to mice maintained on normal diet (ND). (**B**) Effect of HFD on the expression of *Mt1* and *Mt3* gene transcripts in the cerebral white matter. Means ± SEM. Significance was tested using two-tailed Student's t test.

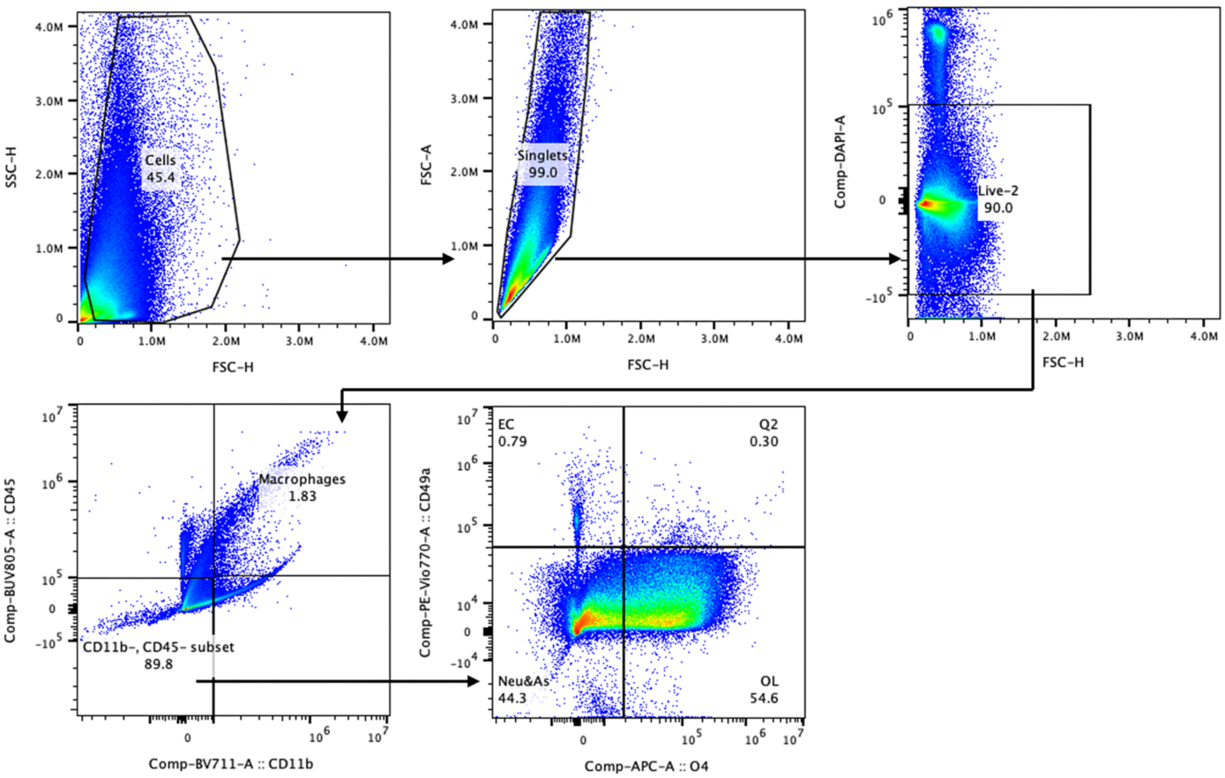

Figure S10. **Gating strategy used to identify macrophages, neural lineage and endothelial cells.** EC, endothelial cells; Neu&As, neurons and astrocytes; OL, oligodendrocytes.

Supplementary Tables

Supplementary Tables 1-5 are provided as separate Excel spreadsheets.

Supplemental Table 6. Age and sex of mice used in each experiment.

Figure	Panels	Genotype	Females		Males	
			N	Age (mo)	N	Age (mo)
Figure 1	A-H	wt	2	2	1	2
		Csf1r+/-	2	2	1	2
Figure 2	A-G	wt	2	2	1	2
		Csf1r+/-	2	2	1	2
Figure 2	H, I	wt	2	2	7	2
		Csf1r+/-	2	2	7	2
Figure 2	J-L	wt	5	3		
		Csf1r+/-	5	3		
Figure 3	A-E	wt	2	2	1	2
		Csf1r+/-	2	2	1	2
Figure 3	F	wt	2	3	2	3
		Csf1r+/-	3	3	1	3
Figure 3	H-L	wt	3	3	2	3
		Csf1r+/-	3	3	2	3
		Csf1r+/-; TgMt1	3	3	2	3
		TgMt1	3	3	2	3
Figure 4		wt	8	>16	10	>16
		Csf1r+/-	11	>16	9	>16
		Csf1r+/-; TgMt1	10	>16	15	>16
		TgMt1	11	>16	7	>16
Figure 5		wt	8	6-7	9	6-7
		Csf1r+/-	6	6-7	8	6-7
		Csf1r+/-; Mt3-/-	8	6-7	4	6-7
		Mt3-/-	9	6-7	2	6-7
Figure 6	A-C	wt	3	>17	4	>17
		Csf1r+/-	3	>17	4	>17
		Csf1r+/-; TgMt1	3	>17	4	>17
		TgMt1	4	>17	3	>17
	D	wt	3	8-9	4	8-9
		Csf1r+/-	6	8-9	2	8-9
		Csf1r+/-; Mt3-/-	4	8-9	4	8-9
		Mt3-/-	6	8-9	2	8-9
Figure 7	A-G	wt	6	>17	7	>17
		Csf1r+/-	5	>17	7	>17
		Csf1r+/-; TgMt1	3	>17	3	>17
		TgMt1	3	>17	4	>17
	J-L	wt young	2	2-3	6	2-3
		Csf1r+/- young	1	2-3	7	2-3
		wt aged	3	>17	6	>17
		Csf1r+/- aged	2	>17	7	>17
		Csf1r+/-; TgMt1 aged	2	>17	3	>17
		TgMt1 aged	2	>17	3	>17
	H,I, M-O	wt	2	8-9	2	8-9
		Csf1r+/-	4	8-9	0	8-9
		Csf1r+/-; Mt3-/-	2	8-9	2	8-9
		Mt3-/-	3	8-9	1	8-9
Figures S1, S3, S8		wt	2	2	1	2
		Csf1r+/-	2	2	1	2
Figure S2		wt	2	2	7	2
		Csf1r+/-	2	2	7	2
Figure S4		wt	15	>16	14	>16
		Csf1r+/-	18	>16	18	>16
		wt_2bAct	14	>16	20	>16
		Csf1r+/-_2bAct	15	>16	12	>16
Figure S5	A-C	wt	9	6-7	13	6-7
		Csf1r+/-	8	6-7	14	6-7
Figure S6	A	wt	3	8-9	4	8-9
		Csf1r+/-	6	8-9	2	8-9
		Csf1r+/-; Mt3-/-	4	8-9	4	8-9
		Mt3-/-	6	8-9	2	8-9
	B	wt	2	8-9	2	8-9
		Csf1r+/-	2	8-9	2	8-9
		Csf1r+/-; Mt3-/-	2	8-9	2	8-9
		Mt3-/-	3	8-9	1	8-9
Figure S7		wt	4	>17	3	>17
		Csf1r+/-	2	>17	5	>17
		Csf1r+/-; TgMt1	3	>17	4	>17
		TgMt1	3	>17	4	>17
Figure S9		wt, ND			6	2.5
		wt, HFD			4	2.5

Supplemental Table 7. Summary of Subjects Analyzed for *MT1, MT3, RPL37* and *RPS29* Gene Expression. Related to Figure 3.

ID	*CSF1R* mutation	Sex	Age	Dur	Neurologic diagnosis	Other medical
CRL1	p.Cys774_Asn814del	M	55	2	FTD	Inflammatory bowel syndrome, hypercholesterolemia, peripheral neuropathy
CRL2	p.Glu633Lys	M	75	2	Atypical Parkinsonism	Hypertension, hypercholesterolemia, hypothyroid
CRL3	p.Met766Thr	M	49	3	NPH (with VPS) versus Binswanger disease	Sleep apnea, hypercholesterolemia, chronic pain
CRL4	p.Pro878Ala	M	64	4	FTD	Sleep apnea, depression
CONT1	NA	M	73	NA	No cognitive impairment	Hypertension, hypercholesterolemia, coronary artery disease, ulcerative colitis
CONT2	NA	F	56	1	Idiopathic myelopathy	Sleep apnea, diabetes, Sjögren's syndrome, schizoaffective disorder
CONT3	NA	M	63	6	Personality disorder, Lewy body dementia	Insomnia, coronary artery disease, COPD
CONT4	NA	M	77	1	MSA	Cardiovascular disease

Abbreviations: CRL = CSF1R-related leukoencephalopathy; ID = case identifier; M= male; F= female; Age = age at death; Dur = disease duration (years); NA = not applicable; FTD = frontotemporal dementia; NPH = normal pressure hydrocephalus; VPS = ventriculoperitoneal shunting; MSA = multiple system atrophy

Supplemental Table 8. Oligonucletides used for qPCR. Related to Figure 3.

**Target**	Orientaton	Sequence
Mouse Mt1	Forward	GCTGTCCTCTAAGCGTCACC
	Reverse	AGGAGCAGCAGCTCTTCTTG
Mouse Mt3	Forward	CTGAGACCTGCCCCTGTC
	Reverse	TTCTCGGCCTCTGCCTTG
Mouse RPL37	Forward	CCAAGCGCAAGAGGAAGTATAA
	Reverse	CATGTCTGAATCTGCGGTAGAC
Mouse RPS29	Forward	GGTCACCAGCAGCTCTACTG
	Reverse	GTCCAACTTAATGAAGCCTATGTCC
Mouse bAct	Forward	AGAGGGAAATCGTGCGTGAC
	Reverse	CAATAGTGATGACCTGGCCGT
Human MT1X	Forward	GCGTGTTTTCCTCTTGATCGG
	Reverse	TTGTCTGACGTCCCTTTGCAG
Human MT3	Forward	CTGAGACCTGCCCCTGCCCTT
	Reverse	TGCTTCTGCCTCAGCTGCCTCT
Human RPL37	Forward	CAAGCGCAAGAGAAAGTATAACTGG
	Reverse	CAGCTGCCCTCTTGGGTTTAG
Human RPS29	Forward	AGGGTTCTCGCTCTTGTCGT
	Reverse	TGCCCCGGATAATCCTCTGAA
Human GAPDH	Forward	ACCCACTCCTCCACCTTTG
	Reverse	CTCTTGTGCTCTTGCTGGG

Supplemental Table 9. Combinations of dyes and antibodies used for flow cytometry. Related to main Figures 2, 6 and 7 and supplementary Figures S2 and S5-S7.

Parameters measured	Cell targets	Antibodies for identification	Dyes*
Labile Zn2+ and Fe2+	Macrophages	CD45 BUV805, CD11b BV711	Zinpyr-1 (detects Zn^{2+})
	Oligodendrocytes	O4 APC	Ferro Orange (detects Fe^{2+})
	Endothelial cells	CD49a PE-Vio770	
	Neurons&Astrocytes	negative selection	
Labile Cu+	Macrophages	CD45 BUV805, CD11b BV711	Cu CF4 (detects Cu^{+})
	Oligodendrocytes	O4 APC	
	Endothelial cells	CD49a PE-Vio770	
	Neurons&Astrocytes	negative selection	
Mitochondrial polarization	Macrophages	CD45 BUV805, CD11b BV711	MitoTracker Red
	Oligodendrocytes	O4 PE	MitoTracker Green
	Endothelial cells	CD49a PE-Vio770	
	Neurons&Astrocytes	negative selection	
ROS, lipid droplets	Macrophages	CD45 BUV805, CD11b BV711	Cell Rox Deep Red
			Mito Sox Red
			Bodipy 493/503

*DAPI (not listed) was included in each sample for gating out dead cells

Supplemental Table 10. Reagents used for flow cytometry. Related to main Figures 2, 6 and 7 and supplementary Figures S2 , S5-S7 and S10.

REAGENT	SOURCE	IDENTIFIER
**Antibodies**		
Anti-CD45 BUV805 (clone 30-F11)	BD Biosciences	Cat#568336; RRID: AB_3684191
Anti-CD11b BV711 (clone M1/70)	BioLegend	Cat#101241; RRID: AB_11218791
Anti-O4 APC (Clone O4)	Miltenyi Biotec	Cat #130-119-155; RRID: AB_2751644
Anti-O4 PE (Clone O4)	Miltenyi Biotec	Cat #130-117-357; RRID: AB_2733887
Anti-CD49a PE-Vio770	Miltenyi Biotec	Cat #130-123-892; RRID: AB_2905052
Anti-mouse CD16/CD32 (clone 2.4G2)	BD	Cat# 553141; RRID: AB_394656
**Chemicals**		
DAPI	Biolegend	Cat# 422801
Zinpyr-1	Cayman Chemical Company	Cat#15122
Copper probe CF4	Target Mol	Cat# T31008
Bio Tracker Ferro Orange	Sigma	Cat# SCT210
Cell ROX Deep Red	Invitrogen	Cat# C10422
Mito SOX Red	Invitrogen	Cat# M36007
MitoTracker Green FM	Invitrogen	Cat# M46750
MitoTracker Red CMXRos	Invitrogen	Cat# M46752
